# AMR Surveillance in low and middle-income settings - A roadmap for participation in the Global Antimicrobial Surveillance System (GLASS)

**DOI:** 10.12688/wellcomeopenres.12527.1

**Published:** 2017-09-26

**Authors:** Anna C. Seale, N. Claire Gordon, Jasmin Islam, Sharon J. Peacock, J. Anthony G. Scott

**Affiliations:** 1 College of Health and Medical Sciences, Haramaya University, Harar, Ethiopia; 2 London School of Hygiene & Tropical Medicine, London, WC1E 7HT, UK; 3 KEMRI-Wellcome Trust Research Programme, Kilifi, Kenya; 4 Nuffield Department of Medicine, University of Oxford, Oxford, OX3 7BN, UK; 5 Department of Medicine, University of Cambridge, Cambridge, CB2 0QQ, UK; 6 Wellcome Trust Sanger Institute, Cambridge, CB10 1SA, UK

**Keywords:** antimicrobial, antibiotic resistance, drug, infection, surveillance

## Abstract

Drug-resistant infections caused by bacteria with increasing antimicrobial resistance (AMR) threaten our ability to treat life-threatening conditions. Tackling AMR requires international collaboration and partnership. An early and leading priority to do this is to strengthen AMR surveillance, particularly in low-income countries where the burden of infectious diseases is highest and where data are most limited.

The World Health Organization (WHO) has developed the Global AMR Surveillance System (GLASS) as one of a number of measures designed to tackle the problem of AMR, and WHO member states have been encouraged to produce National Action Plans for AMR by 2017. However, low-income countries are unlikely to have the resources or capacity to implement all the components in the GLASS manual. To facilitate their efforts, we developed a guideline that is aligned to the GLASS procedures, but written specifically for implementation in low-income countries. The guideline allows for flexibility across different systems, but has sufficient standardisation of core protocols to ensure that, if followed, data will be valid and comparable. This will ensure that the surveillance programme can provide health intelligence data to inform evidence-based interventions at local, national and international levels.

## Introduction

Antimicrobial resistance (AMR) develops when strains of micro-organisms evolve to survive exposure to antimicrobial agents. The subsequent transmission and spread of resistant pathogenic bacteria sets the scene for development of drug-resistant infections (DRIs). The increasing use of antimicrobials worldwide has been associated with a global increase in DRIs, which threatens to return clinical therapies to the pre-antibiotic era. At present, DRIs are estimated to account for 50,000 deaths each year in Europe and the USA alone ^1^, but by 2050 it is estimated that DRIs will account for 10 million deaths per year worldwide, posing an economic and biosecurity threat ^2^.

Countries with the highest burdens of communicable diseases usually have the least resources and, in these settings, data on AMR and DRIs are most limited ^3, 4^. While large regional AMR surveillance networks have been established in Europe (EARS-Net), Latin America (Red Latinoamericana de Vigilancia de la Resistencia a los Antimicrobianos, ReLAVRA) and Central Asia and Eastern Europe (CAESAR), capacity for AMR surveillance in low-income countries is relatively limited and fragmented, despite evidence that, as with the rest of the world, AMR in low-income regions is increasing ^3^.

The importance of strengthening AMR surveillance in low-income countries was highlighted in 2014 by a United Kingdom government-commissioned review ^1^. In response, the United Kingdom Department of Health launched the Fleming Fund to support low-income countries in developing AMR surveillance systems. The fund is aligned with the World Health Organization (WHO)’s Global AMR Surveillance System (GLASS) ^5^ to support the Global Action Plan on AMR ^3^. The aims of the WHO AMR surveillance programme include monitoring trends in infection and resistance to develop standard treatment guidelines that support best practice for patient care, but also recognise the importance of linking information on AMR from different sectors, such as human, animal, food, agriculture, environment, and data on antibiotic use in human and animal populations and environmental antibiotic usage. AMR surveillance should also allow for assessment of interventions to reduce AMR, provide early alerts for emergence of novel resistant strains, and aid the rapid identification and control of outbreaks ^6^.

Recognising that AMR surveillance capacity varies considerably, to facilitate AMR surveillance and participation in GLASS for low-income countries, we aimed to identify an approach to allow independent development of each component of surveillance to build a comprehensive system. This result is a full guideline ( Supplementary File 1) that has been developed with the objective of supporting capacity development in a standardised manner while allowing flexibility to incorporate country or regional priorities. The guideline is intended to:

- be suitable for use by low-income countries, recognising the context of different health systems;
- be based on an assessment of available evidence and review of established protocols in comparable resource settings;
- provide a basis for early collection and analysis of data on AMR that will help countries to assess the extent of AMR in important pathogens and participate in global and regional surveillance (GLASS);
- take into account the need for epidemiological and statistical validity and quality assurance, so that the data can be used, shared and combined to provide reliable evidence of AMR prevalence and to evaluate the effectiveness of interventions;
- provide a tiered structure, with a minimum level of essential (core) activities and scope for expansion so that countries can select the level of operation to suit their circumstances, with the ability to expand and broaden to advanced surveillance activities over time;
- provide a roadmap for improving laboratory capacity, data collection and surveillance for AMR with an effective One Health approach, through multi-sectoral involvement across the interface between humans, animals and their various environments.

While recognising the global importance of drug resistance among viruses, fungi and parasites, this document focuses on bacterial infections in humans, and particularly on eight pathogens identified by the WHO as priority organisms for the early implementation of AMR surveillance. However, we anticipate that activities that improve the isolation, identification, susceptibility testing and reporting of these organisms will support development of clinical diagnostics for other pathogens, and can be tailored in-country for locally important or emergent bacteria.

Similarly, while the emphasis in this guideline is on human clinical pathogens, we recommend, in line with WHO, that AMR surveillance, in the long term, be embedded in a One Health approach. To support this, there should be multi-sector representation (including involvement from agriculture and veterinary medicine) in AMR surveillance bodies from the outset, in order to inform, monitor and control the threat to public health arising from AMR.

## Methods

We brought together a team with expertise in microbiology, genomics, epidemiology, public health, infectious diseases and experience in setting up surveillance systems in low income countries. We reviewed the existing published and grey literature on infectious disease surveillance networks, including the Global Antibiotic Resistance Partnership (GARP), European Antimicrobial Resistance Surveillance Network (EARS-Net), Latin American Antimicrobial Resistance Surveillance Network (ReLAVRA), Central Asian and Eastern European Surveillance of Antimicrobial Resistance (CAESAR), and the Worldwide Antimalarial Resistance Network (WARN). These were critically assessed according to the context they were designed for, and their relevance to AMR surveillance in low and middle income countries. Concurrently, we reviewed AMR surveillance systems and existing AMR surveillance capacity ^7^.

Based on these data, and aligned to the Global Antimicrobial Surveillance System (GLASS), we drafted proposals for a roadmap for participation in GLASS. We shared these with experts and stakeholders in a two-day meeting in London, United Kingdom (July 2016). The meeting included representatives from the WHO, The Wellcome Trust, the Bill & Melinda Gates Foundation, Public Health England, and representatives of relevant networks such as the Central Asian and Eastern European Surveillance of Antimicrobial Resistance (CAESAR). We included expertise and representation from a range of key geographies in sub-Saharan Africa and South Asia. Following this, we revised our proposals, and recirculated for final review. The full guideline is included as Supplementary File 1 to this article.

## Results

### AMR Surveillance system

We recommend a sentinel surveillance system ^8^, with step-wise expansion to increase the numbers of participating sites and their scope. In the first instance, we propose that countries should identify or develop capacity in a single site that can undertake surveillance to an acceptable core standard. Having achieved that standard, the primary site should support the development of good practice in secondary sites, with the long-term aim of building a comprehensive network of sentinel sites which can provide high-quality representative AMR data. Sentinel sites that have achieved core capacity may aspire to higher standards (extended and advanced, Supplementary File 1: Appendix D) by developing and extending their capabilities.

### Legal and ethical considerations

Public health surveillance is usually legally mandated by the national government. For public health surveillance programmes, the probability and the magnitude of harm to the population arising from not reporting surveillance data must be moderate to major to justify the use of individual patient data without individual patient consent ^9^. In this context, the WHO has recently recognised AMR as a significant potential global health threat ^10^. Reporting the characteristics of resistant pathogens rarely represents a threat to patient confidentiality, but the inclusion of simple clinical data such as age, sex, collection date, specimen type and syndromic diagnosis adds considerable value to the information obtained from the laboratory, and there are clear benefits from AMR surveillance at patient, pathogen and population levels ^6^. Examples of the application of AMR data include timely feedback to clinicians to support patient care and enable rationalisation of antibiotic treatment; use of data to inform local antimicrobial prescribing guidelines and infection control policies; analysis of clinical surveillance data (at international, national and / or local level) to enable public health interventions; cross-policy collaboration and support for research institutions to analyse clinical surveillance data, adopting a One Health to understand the emergence, transmission and dissemination of pathogens at the human-animal interface.

Given the need to integrate data from different sources, including individual patient data, it is essential that there are data governance agreements and procedures in place. These should protect the confidentiality of individual patients, but also facilitate the sharing of AMR surveillance data to inform policy locally, nationally and internationally. To meet ethical obligations, technical, legal and/or political barriers to data sharing ^11^ must be overcome, and best practice for data collection ensured. For these reasons, a successful AMR surveillance programme requires clear political support, and should engage accordingly with the relevant government bodies ^11^.

### National Action Plan

The first step in establishing AMR surveillance is the development of a National Action Plan (NAP) for AMR, as set out by the WHO Global Action Plan on AMR ^3^. WHO member states have been encouraged to develop NAPs and a manual and template are available to support this process ( http://www.who.int/drugresistance/action-plans/manual/en/).

### Governance and structures

**Figure 1.**
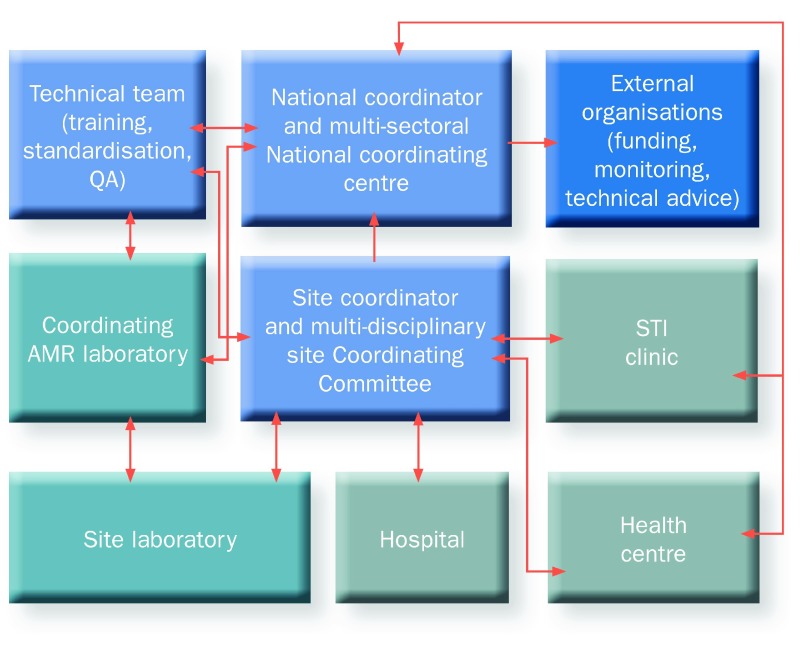
Example organizational structure for AMR Surveillance in low resource settings. AMR, antimicrobial resistance; QA, quality assurance.

Each country should develop its own organisational structures ( Figure 1), and define terms of reference. While the governance structure may vary, important factors include identification of a National Coordinating Centre (NCC), convening a technical team, and strong engagement with the Ministry of Health, reflecting the national importance of AMR surveillance in health systems.

The NCC should include a committee of multi-sectoral stakeholders to support a One Health approach at national and international levels. This committee could be, or could develop from, the national working group on AMR as established by GARP, or the committee responsible for the NAP. The committee should report to the appropriate national body, for example, the Ministry of Health, and, where appropriate, collaborate with a relevant external organisation/funder.

The roles and responsibilities of the committee should be set out with formal terms of reference. Membership should include relevant technical experts and stakeholders, although individuals may fulfil the remit of more than one technical brief. A typical committee may include the following representatives: technical team leader, Ministry of Health, Ministry of Agriculture, national public health institute, coordinating AMR laboratory, international stakeholders, clinical microbiologist, data manager, public health analyst, laboratory manager, hospital manager, adult physician, paediatrician, pharmacist, veterinarian, infection control manager.

The functions of the NCC include:

- commissioning a situational analysis of current capacity and sustainability for AMR surveillance
- national strategic planning for AMR surveillance
- oversight of AMR surveillance implementation at a national level against key performance indicators

The strategic function may be extended to include other aspects of tackling AMR, for example strategic oversight of infection prevention and control (IPC) policy, development and use of standardised treatment guidelines, and control of the sale of antimicrobial agents.

The NCC should have oversight of the technical team to: monitor quality assurance, support capacity building through training of national and site level participants, determine national priorities for pathogens in AMR surveillance in addition to those identified as priority pathogens by the WHO, review the scope of AMR surveillance as capacity develops, integrate a One Health approach, review the introduction of new technologies, support research programmes to use AMR surveillance platforms, collaborate with neighbouring countries and across international regions, and develop and expand regional networks.

The NCC should be headed by a named National Coordinator for AMR surveillance from a key stakeholder institution, such as the Ministry of Health, Institute of Public Health, or similar organisation. The National Coordinator should be supported by a technical team responsible for training, standardisation and quality assurance. Where appropriate, the technical team may be led by the National Coordinator.

### External organizations

The NCC should collaborate with international stakeholders and funding bodies, such as the Fleming Fund, the US Centers for Disease Control and Prevention, the Institut Pasteur, the European Centre for Disease Prevention and Control, the Bill & Melinda Gates Foundation, and major non-governmental organizations including Médecins sans Frontières, the Global Health Security Agenda and the WHO.

The NCC should work with external bodies to ensure standardisation, training and internal and external quality assurance of all processes relating to AMR surveillance across participating countries, for example by developing and participating in national and international workshops.

### Site Coordinating Committee

Sentinel sites should determine and define their own organizational structures, and how this fits into existing hospital and laboratory administration systems. There should, however, be a Site Coordinating Committee (SCC), with defined terms of reference, and which includes relevant representatives, for example site leader, hospital administrator, data manager, laboratory manager, clinical microbiologist, adult physician, paediatrician, infection control manager, pharmacist, veterinary practitioner, public health specialist.

The site leader would be expected to have project management and programme implementation skills, and should report to the NCC. The role of the SCC, led by the site coordinator, includes:

- working with the national technical team to facilitate a situational analysis of current capacity and sustainability at the site
- planning strategic priorities at the site
- oversight of AMR surveillance implementation at the site and reporting against key indicators

The roles of the SCC are to support on-site training for AMR procedures, develop locally-adapted standard operating procedures (SOPs), ensure quality control measures and regular audit for all AMR surveillance processes, work with the national technical team to establish internal quality assurance assessment (and ultimately progress to external quality assurance assessment), ensure effective lines of communication are in place for feedback of AMR results to clinicians, feedback of summarised AMR data to local participants and stakeholders (administration, clinical, laboratory and data staff), and report anonymised case-level data to the National Coordinator in a timely manner. The strategic function of the SCC may be extended to include other aspects of tackling AMR, for example, ensuring nationally agreed infection prevention and control policies and treatment guidelines are being followed.

### Laboratories

A coordinating AMR laboratory should be identified / established for AMR surveillance. This may already be in place, or may be developed as part of the capacity-building process. Where there is no capacity for a coordinating AMR laboratory, countries should collaborate with neighbouring countries, which may be able to provide this service.

Coordinating AMR laboratories should be accredited, or be working towards laboratory accreditation ^12^. Their staff should be trained by the technical team and / or external partners to provide training for sentinel site laboratory staff, using a “Train the Trainers” approach ( Supplementary File 1: Appendix A). The functions of the coordinating AMR laboratory are:

1) core laboratory processes ( Supplementary File 1: Appendix D)
2) participation in internal quality assurance
3) participation in external quality assurance through appropriate international schemes
4) provision of a reference service for core organism / antimicrobial combinations as a minimum, for borderline isolates or isolates with unexpected or unusual resistance profiles, and collaboration with international centres to monitor emerging resistance patterns
5) assisting sentinel site laboratories to procure equipment and reagents, in collaboration with the NCC
6) maintaining a biorepository for bacterial isolates
7) promotion of good practice (including development of national SOPs) to ensure standardisation and quality control
8) training staff at sentinel site laboratories
9) facilitating the development of internal quality assurance at sentinel site laboratories
10) provision of external quality assurance across sentinel site laboratories if they do not already participate in external quality assurance (EQA) (for example, by testing a subset of isolates from the sentinel site laboratories and providing feedback)

Each sentinel site should have its own laboratory, or access to a laboratory, which is able to:

1) provide core laboratory processes, including isolate identification, susceptibility testing and storage ( Supplementary File 1: Appendix E)
2) communicate AMR results (organism and susceptibilities) to clinicians in a timely manner to improve the care of individual patients
3) refer isolates with unusual, unexpected or indeterminate resistance patterns to the coordinating AMR laboratory for further testing
4) participate in on-site training and attend national training as appropriate
5) adhere to localised SOPs with quality control checks
6) conduct internal quality assurance procedures
7) work with the technical team and coordinating AMR laboratory to develop capacity, working towards participation in EQA and gaining laboratory accreditation

### Situational analysis

A situational analysis of AMR should be undertaken nationally. This should consider all aspects of AMR surveillance, including clinical sampling, laboratory procedures and data systems. A detailed laboratory assessment can be performed using the WHO’s Laboratory Assessment Tool ( Supplementary File 1: Appendix A).

### Training

To promote awareness of AMR surveillance, education and training should be integrated into local and national education programmes, across all the disciplines required for AMR surveillance. These include clinical, laboratory, information technology and public health training ( Supplementary File 1: Figure 3). Teaching on AMR should be introduced into formal training pathways, including undergraduate and postgraduate curricula across these disciplines. AMR awareness should also be developed through continuing professional development (training days, workshops) at site, regional, national, and international levels. Such training should incorporate e-learning options and specific training modules. To enhance motivation, site coordinating committees should consider appointing individuals with specific roles to act as AMR surveillance champions in clinical (doctors, nurses or allied professions) including infection prevention and control, laboratory and data services.

### Sentinel site identification

The initial situational analysis should identify potential sites for AMR sentinel site surveillance. Site selection should be undertaken by the NCC through a transparent process, with involvement of an external stakeholder or funder where appropriate.

The sites selected, and the network as a whole, should reflect relevant variations in geography, socioeconomic factors and demography, disease epidemiology (e.g. co-morbidities such as HIV) and ecology, taking into account climate, rainfall and land use.

Surveillance that only represents one level of healthcare (e.g. referral hospitals) will not adequately reflect the AMR situation of the country. The potential for biases include:

1) sampling only from referral hospitals, which may have high numbers of patients treated with antibiotics prior to sampling or high numbers of cases who have failed first-line treatment at referring facilities
2) sampling only from hospitals may under-represent less severe infections e.g. sexually-transmitted infections, uncomplicated urinary tract infections, community acquired pneumonia.
3) sampling only from healthcare outpatient clinics will result in under-representation of severe or invasive infection
4) health financing systems that require patients to pay for investigations will include only those who are able to afford investigations

AMR surveillance sampling should therefore be drawn from the health facilities used by the population targeted for surveillance. These may include referral hospitals, district hospitals and out-patient facilities (including primary care); some institutions may fulfil more than one of these functions. Facilities serving a population sub-group, such as private hospitals in a country where most hospital services are delivered through the public sector, should only be included if the rest of the population is already adequately represented.

It is anticipated that sites and settings will be identified with very different levels of capacity ( Supplementary File 1: Box 1). At the initiation of AMR surveillance it is important to identify organisational and leadership strengths in order to develop systems and technical capacity. Key factors to consider when evaluating the potential of individual sentinel sites are:

- whether the site has capacity and support (from local management / government / populations) to connect to a district or national network and subsequently share data with international agents, including the WHO
- whether the site will be able to contribute to the national network through mentoring and supporting capacity building at subsequent sites
- what level of investment will be required to achieve and sustain core AMR surveillance participation

Once a site has been identified as a potential AMR sentinel surveillance site, a more detailed technical analysis should be performed to determine which core / extended / advanced ( Supplementary File 1: Appendix E) activities are being performed to the required standards, and what investments are required to facilitate full participation in surveillance.

### Levels of AMR surveillance

To reflect variation in capacity between countries and regions, core, extended and advanced functions of AMR surveillance are described here, with the aim of prioritising a core standard to ensure basic quality data ( Table 1 and Table 2). When these core processes are functioning at acceptable standards, sentinel sites should consider extending their capacity to include extended, and / or advanced functions, and to support other sites to develop their capacity.

**Table 1.** National functions for antimicrobial resistance (AMR) Surveillance (core, extended, advanced).

AMR surveillance component		Requirements and standards for core level	Extended level activities *	Advanced level activities **
**Overall aim**		Surveillance data drive national policy and international policy		
**Leadership**	***Data analysis***	National coordinating centre reviews aggregated data with annual report	National coordinating centre reviews aggregated data quarterly with annual report	Real time data presentation (dashboards) Surveillance data are compared to modelled estimates to assess the emergence of resistance and provide early warning for public health action
	***Data*** ***governance***	National standards for data governance and data sharing agreements		
	***Assessment of*** ***evidence***	National coordinating centre reviews aggregated data with expert advice where needed	National coordinating centre liaises with regional network	
	***Intervention***	Surveillance data drive national policy and international policy		
**Training**	***Clinical***	Training programmes for key staff in core clinical surveillance procedures	Established national training programmes using diverse platforms (e.g. electronic media). Integration of AMR into relevant (undergraduate and postgraduate) programmes.	Functions as a regional centre for international training programmes. Adapts training materials for international use (translation, electronic training packages in different languages)
	***Laboratory***	Training programmes for key staff in core laboratory surveillance procedures	Established national training programmes using diverse platforms (e.g. electronic media) Integration of AMR into relevant (undergraduate and postgraduate) programmes.	Functions as a regional centre for international training programmes. Adapts training materials for international use (translation, electronic training packages in different languages)
	***Data***	Training programmes for key staff working in surveillance sites in core data surveillance procedures	Established national training programmes using diverse platforms (e.g. electronic media)	Functions as a centre for international training programmes. Adapts training materials for international use (translation, electronic training packages in different languages)
**Quality assurance** **(QA)**	***Clinical***	Annual site visit and audit of clinical surveillance at sentinel sites by technical team and national coordinator	Quarterly external audit of clinical data submitted through automated systems and comparison with other sites	
	***Sentinel site*** ***laboratory***	Annual site visit and audit of laboratory standards by technical team and national coordinator	QA assessment of laboratory site to international standards with external accreditation	
	***AMR laboratory***	Coordinating AMR laboratory participating in external QA and providing internal QA to site laboratories	Coordinating AMR laboratory performs extended testing (e.g. minimum inhibitory concs.) on a subset of isolates. Collaborates with external partners to investigate exceptional resistance patterns (including whole genome sequencing).	Provision of whole genome sequencing (WGS) for isolates of interest
	***Data***	Annual site visit and audit of data systems by technical team and national coordinator	Support for automated sharing of site data for national aggregation	
**Coordinating AMR** **laboratory**	***Storage of*** ***isolates***	Freezer storage (-20°C) of resistant isolates with linkage to paper or electronic database	Reliable freezer storage (-80°C) of resistant isolates with linkage to electronic database ^#^	
	***Transport to*** ***AMR laboratory***	Invasive isolates are transferred to AMR laboratory annually according to local standard operating procedures at acceptable biosafety standards	Invasive isolates are transferred to AMR laboratory quarterly and according to acceptable biosafety standards	

*All core process are assumed in the extended level

**All core and extended processes are assumed in the advanced level

^#^Or other innovative method such as freeze-drying, as used at the Oxford University Clinical Research Unit, Vietnam.

**Table 2.** Sentinel site functions for antimicrobial resistance (AMR) Surveillance (core, extended, advanced).

AMR surveillance component		Requirements and standards for core level	Extended level activities	Advanced level activities
**Overall aim**		Surveillance data inform individual care	Surveillance data drive local and national policy (e.g. empiric treatment guidelines, drug procurement) and public health activities	
**Clinical admission** **assessment and** **investigation**	***Clinical admission*** ***assessment***	Clinical history and examination and investigation based on physician (syndromic) diagnosis.	Systematic clinical history and examination according to clinical algorithms in all patients presenting with suspected infection.	Standardised admission proforma documenting clinical signs and symptoms used to guide diagnosis.
	***Clinical data***	Clinical data included in (paper) request for laboratory investigation, with unique alphanumeric identifier	Clinical data included in (electronic) request for laboratory investigation, with unique alphanumeric identifier	Linkage of extended clinical data (e.g. vital signs, blood results, outcomes) with laboratory data
	***Clinical*** ***investigation***	Systematic investigation based on physician syndromic diagnosis	Systematic investigation based on clinical findings.	
	***Training and*** ***quality assurance***	Routine training for surveillance standard operating procedures (SOPs), quality control and Internal Quality Assessment.	External Quality Assessment	Functions as a regional training centre
**Isolate identification** **and susceptibility** **testing**	***Sample transport***	Samples transported according to local SOPs	Samples transported according to international biosafety standards	
	***Sample*** ***registration***	Local laboratory paper based data system	Electronic laboratory data system	
	***Culture and*** ***identification***	Automated blood culture system and capacity to identify the relevant priority pathogens according to SOPs	Automated blood culture; CSF, urine, stool and swab culture, identifying all isolates according to SOPs for all priority pathogens.	Automated identification (e.g. MALDI- TOF)
	***Susceptibility*** ***testing***	Use of disc diffusion for blood culture priority pathogens according to SOPs	Use of disc diffusion methods according to SOPs for all species; may include e-tests or broth dilution methods.	Automated identification (e.g. VITEK)
	***Training and*** ***quality assurance*** ***(QA)***	Routine training for SOPs, quality control and internal QA	External quality assessment	Functions as a regional training centre
**Isolate storage** **(local) and referral to** **AMR laboratory**	***Storage of*** ***isolates***	Freezer storage (-20°C) of resistant isolates with linkage to paper or electronic database	Reliable (generator back-up) freezer storage (-80°C) of resistant isolates with linkage to electronic database	
	***Transport to AMR*** ***laboratory***	Invasive isolates are transferred to AMR laboratory annually according to SOPs	Invasive isolates are transferred to AMR laboratory quarterly according to international standards for biosafety	
	***Training and QA***	Routine training for isolate storage, SOPs, quality control and internal quality assessment	External quality assessment	
**Data review**	***Data use***	Anonymised individual data submitted to national coordinating centre and shared regionally and internationally		Automated real time submission of data to national network
	***Data linkage***	Clinical and laboratory data linked by recording them on the same lab request form	Automated linkage between clinical request data and laboratory data	Automated linkage between clinical and laboratory databases
	***Data governance***	Data sharing policy and agreements in place in collaboration with the Ministry of Health and/or national public health institute		

The choice of target level of surveillance should depend on:

- Current in-country capacity in clinical, laboratory and data handling areas
- Start-up and ongoing costs of the proposed AMR surveillance system
- Sustainability of the proposed AMR surveillance system

### Technical components

**Figure 2.**
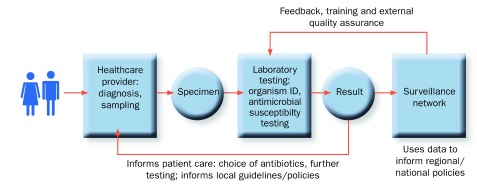
Antimicrobial resistance Surveillance process.

To allow full and informative interpretation of data, effective AMR surveillance requires well-functioning health-systems that serve a defined population. Standard laboratory methods for pathogen identification and antimicrobial susceptibility testing are vital in order to understand the emergence of AMR and inform policy, but so too are population descriptors, healthcare utilisation patterns, and the systematic assessment and investigation of patients ( Figure 2).

***Population catchment and sampling frame.*** Wherever possible, the catchment population of the health facilities included in surveillance should be defined and an assessment should be made of the patterns of healthcare utilisation in that population. This is important for data interpretation: total population allows estimates of incidence and trends; descriptors define risk factors (socio-economic status, urbanisation, co-morbidity levels) for national models of AMR burden; access to care patterns determine whether the healthcare facilities included are the first point of contact, post-treatment, or post-clinical failure level – which will have different AMR prevalence. Health facility selection is an important part of sentinel site selection and a sentinel site laboratory should receive samples from both inpatient and outpatient clinic facilities, with costs associated with AMR surveillance covered at an institutional or national level, rather than directly charged to patients.

At the extended level, a healthcare utilisation survey would be appropriate, and at an advanced level the population catchment should be described using census data or by an enumeration survey. It may also be appropriate to make use of existing Health and Demographic Surveillance Systems (HDSS) ^6^.

***Clinical surveillance.*** AMR surveillance data should be interpreted in the context of local clinical practice. This is particularly relevant for low-income country settings, which use syndromic management approaches where patients are diagnosed clinically and treated empirically.

At a core level, the clinical data on the laboratory request form should include the clinical diagnosis selected from a list of syndromes. For adults this includes sepsis, severe pneumonia, acute diarrhoea, bacterial meningitis, severe soft tissue infection, pyelonephritis, sexually transmitted infections ( Supplementary File 1: Appendix B) or other (to allow for clinical discretion). The clinical syndromes for children include severe diarrhoeal disease, severe febrile illness, meningitis, severe pneumonia and neonatal possible serious bacterial infection ( Supplementary File 1: Appendix C).

At an extended level, clinical assessment of adults and children (<5 years) should be based on standardised and systematic history and examination with case definitions from national and international guidance ( Supplementary File 1: Appendices B and C) ^13^. At an advanced level, diagnosis should be supported by clinical proformas with electronic storage of these extended clinical data (to be electronically linked to laboratory data).

Clinical sampling for AMR surveillance should be guided by the syndromic diagnosis for which the patient is being treated (see Supplementary File 1: Appendices B and C for suggested outline), with additional investigations undertaken at the clinician’s discretion. This supports interpretation of the data to guide empiric therapies and reduces potential bias, which may occur if only clinical treatment failures or the most seriously ill patients are investigated.

Blood culture is the core sample for AMR surveillance, as an indicator of pathogens causing severe, invasive and life-threatening disease. It is anticipated that sentinel site laboratories will also process other samples, but capacity building and data collection should initially focus on blood cultures as a core function. Once blood cultures are collected and processed to an acceptable standard, the laboratory should be encouraged to focus on cerebrospinal fluid (CSF) as the next priority sample associated with serious disease. At the extended level, laboratories should also have capacity to process urine, stool and urethral / cervical swabs to AMR surveillance standards.

Appropriate staff training and SOPs should be in place for all procedures including collection, transport, registration, processing and reporting of samples. Personal protective equipment should be available, and sample transport should be undertaken safely, securely and in a timely fashion (see Supplementary File 1: Appendix A for links to safety manuals and guidance documents).

***Isolate identification.*** Specimen culture and testing for antimicrobial susceptibility should be done by sentinel site laboratories. Isolates with unusual susceptibility profiles, or of uncertain identification, should be referred to the coordinating AMR laboratory, as well as a proportion of all isolates for quality control purposes. All isolates from blood or CSF specimens should be sent to the coordinating AMR laboratory for storage.

Reporting for AMR surveillance should focus on the eight WHO priority pathogens as described in the GLASS manual ( *Escherichia coli, Klebsiella pneumoniae, Acinetobacter baumannii, Staphylococcus aureus, Streptococcus pneumoniae, Salmonella* spp. *, Shigella* spp. and *Neisseria gonorrhoeae)* and other pathogens of local or national importance *.*

At core level, pathogen identification should be done by using relevant biochemical and / or serological tests ( Supplementary File 1: Appendix F). At the advanced level, laboratories may use molecular methods and automated systems such as MALDI-TOF, Vitek or Microscan ( Supplementary File 1: Appendix E).

***Antimicrobial susceptibility testing.*** AMR surveillance programmes should include, but should not be limited to, the following bacteria-antimicrobial drug combinations in compliance with the GLASS manual (see Supplementary File 1: Appendix G for all combinations) ^14^:

- *Escherichia coli* vs. 3rd generation cephalosporins and fluoroquinolones;
- *Klebsiella pneumoniae* vs. 3rd generation cephalosporins and carbapenems;
- *Staphylococcus aureus* vs. oxacillin or cefoxitin;
- *Streptococcus pneumoniae* vs. penicillin or oxacillin;
- *Salmonella* species vs. fluoroquinolones;
- *Shigella* species vs. fluoroquinolones;
- *Neisseria gonorrhoeae* vs. 3rd generation cephalosporins

Antimicrobial susceptibility testing for priority pathogens should be carried out in line with international standards, preferably according to the European Committee on Antimicrobial Susceptibility Testing (EUCAST) methodology and guidance ( www.eucast.org). Where Clinical and Laboratory Standards Institute (CLSI) guidelines are used, these may also be reported. Unless automated systems are already in place, antimicrobial susceptibility testing at the core level should be performed using the disc diffusion method. Where additional drugs are included (for example *Acinetobacter baumannii* vs. carbapenems), they should be tested according to accepted guidelines (e.g. CLSI, EUCAST).

Sentinel site laboratories should document whether isolates are susceptible, intermediate or resistant (S/I/R) according to clinical breakpoints defined by EUCAST or CLSI. Zone sizes (mm) should also be measured and recorded, to allow for retrospective adjustment if new breakpoints are set. At the extended and advanced levels, minimum inhibitory concentrations (MICs) may be determined, e.g. by microbroth dilution (manual or automated) or gradient diffusion tests such as E-Tests. MIC values should be recorded (in case breakpoints change in the future).

***Data management.*** Reporting results requires efficient data management at both sentinel site and national levels ( Supplementary File 1: Figure 5). Quality control should be incorporated at every stage, with automated data validity checks and rules, as well as audit to check data consistency, completeness and accuracy. Confidentiality should be protected and data security measures should be in place (links to resources are given in Supplementary File 1: Appendix A).

The site coordinator should ensure individual case-level anonymised data (as set out below) are submitted to the national coordinator with health facility data. These include the total number of patient episodes and the total number of samples processed in the laboratory. The site coordinator should feedback sentinel site data at least quarterly to healthcare administration, clinical and laboratory staff, to support continued engagement with AMR surveillance.

At core level, clinical data should be recorded in a standardised paper request form that accompanies the clinical sample to the laboratory. Sites operating at extended level will capture data using an electronic system. The minimum set of data required on the core clinical request form are: age, sex, clinical diagnosis, specimen type, sample date, admission date, hospital or community source. Data fields collected at the extended level include: healthcare facility type (referral, district, health centre, general clinic, and STI clinic), admission ward, initial antimicrobial treatment and clinical diagnosis (with specific clinical signs and symptoms recorded at an advanced level). At core level, clinical and laboratory data should be linked through use of a single paper form on two sides of the same piece of paper – and can be entered (double entered to avoid transcription errors) electronically for onward transmission, at the end of the processing. Unique specimen numbers should be assigned to each sample, as well as a unique alphanumeric identifier for the patient episode.

Laboratories should routinely keep records of and report all investigations carried out, including those that are negative. For surveillance purposes, only the first isolate for each patient (per quarter) should be included for AMR surveillance reporting. Systematic reporting of data is important to reduce the bias that arises if resistant organisms are over-reported, or reported only if resistant to certain antibiotics.

Sites operating at extended level will capture laboratory data in an integrated electronic system such as WHONET (see link provided in Supplementary File 1: Appendix A). Clinical and laboratory data should be linked through the unique specimen identification number. WHONET has been developed to facilitate AMR surveillance reporting: but other systems can be used and data specification for aggregated data upload to the GLASS IT platform is available.

***Use of innovative technologies and mobile communications.*** In high-income settings, innovative technologies for diagnostics, therapeutics and data management are integrated into most health systems, with funding streams for research and executive bodies to evaluate and approve new technologies. In low-income countries, WHO and other bodies provide support for the implementation of new technologies, and these should be considered by countries developing AMR surveillance ^14^. Examples of innovative technologies relevant to laboratory diagnostics under assessment include ^15^:

- mobile phone systems for sending microscope images – this could be extended to use of smartphones to share or assess images of disc diffusion assays to confirm zone size
- use of electronic health records
- nucleic acid amplification for TB diagnostics – these technologies could be developed to allow identification of resistance by genetic rather than culture methods, with options for cloud-based reporting
- solar-powered autoclaves
- freeze-drying bacterial isolates for storage (vs freezing at -80°C)

### Monitoring, evaluation and development

***Quality assurance.*** Quality assurance (QA) should be led by the national coordinator and technical team in country, in conjunction with external organizations as appropriate. At a core level, all site procedures should be undertaken according to SOPs, adapted from national SOPs, and based on these guidelines. Alongside these, quality control (QC) and QA procedures should be established to ensure that the data produced are accurate and reliable.

In a clinical setting, standardisation and investigation should be assessed through standard quality control procedures, ensuring completeness of the data and investigations requested through audit assessment and feedback. To do this, hospital level data on all admissions are required to assess, for example, the diagnosis of all patients and whether those with an infectious syndrome were appropriately investigated. At a core level, the quality of clinical sampling and the data acquired should be subject to internal quality assurance assessment through the national coordinator and technical team. At the extended level, external assessment would be expected through an independent monitor.

Laboratory QA involves in-house quality control procedures, and internal QA and external QA (EQA) assessment. QA measures include specimen collection and transportation (e.g. transport times, specimen quality); the performance of test procedures, reagents, disks used, media, instruments, and personnel, and test results and documentation. EQA is a system for validating laboratory performance using an external, objective agency. EQA is essential for accredited laboratories and, where possible, all laboratories should participate in a formal EQA scheme for all tests performed. Traditional proficiency testing is considered to be the most cost-effective and useful EQA method. This involves regular (at least annual) dispatch of test isolates to laboratories, to be processed using the normal testing methods by staff who routinely handle such samples. Results are submitted to a central agency, which provides feedback and allows comparison with results from other laboratories (schemes listed in Supplementary File 1: Appendix A). If participation in formal proficiency testing is not possible, adequate EQA may achieved through a combination of within country retesting / rechecking and internal quality assurance and control procedures, with periodic external observation of practices and procedures by qualified personnel. This function could be provided by the coordinating AMR laboratory. All laboratories should be engaged in quality improvement (e.g. using the WHO Laboratory Assessment Tool), and should be encouraged to work towards full accreditation (e.g. WHO Stepwise Laboratory Improvement Process Towards Accreditation in the African Region (SLIPTA); see links provided in Supplementary File 1: Appendix A).

Data systems and data management processes should include standard QC measures as described. They should also be subject to internal and external quality assessment by the National Coordinator and Technical Team (internal) and an external monitor. Evaluation should be through comparison of the data system description, the data dictionary and the data report from each site with those from other sentinel sites and other country systems.

### Key Performance Indicators

Key Performance Indicators (KPIs) are used to monitor progress and identify sentinel sites where problems are arising and more detailed investigation is needed to understand why the indicators are not being met. The purpose of this investigation is to support sentinel sites to achieve the KPIs. GLASS is developing a monitoring framework for AMR surveillance and provides a sample framework for national KPIs (see Supplementary File 1: Appendix A for link). In-country indicators should be agreed at the inception of AMR surveillance and reviewed annually by the NCC.

Sites will vary in terms of population, geography, and health care facility. However, the criteria given in Supplementary File 1: Box 2 illustrate examples of the criteria that a well-functioning AMR surveillance site would be expected to meet.

## Conclusions

Development of AMR surveillance is essential to address the global challenge of DRI. It is expected that, in line with GLASS, AMR surveillance systems will develop in low-income countries to extend AMR surveillance progressively beyond what is described here, to include agriculture (including animal health) and the environment in a One Health approach. These activities were beyond the scope of this work, and are normally conducted by parallel laboratory systems; however, they should be considered by NCCs as the capacity for AMR surveillance in clinical settings advances. Further work is also needed to interpret microbiological data in the context of antibiotic consumption data. This could be done with aggregate data from national wholesale data, or using point prevalence surveys of antimicrobial prescriptions by indication (clinical syndromes), at repeated intervals, for example six-monthly.

The outputs of AMR surveillance must be used to underpin public health policy, locally, nationally and internationally. In addition, where possible, surveillance systems should provide a platform to answer research questions with local, national and international collaborations, which will inform our understanding of the emergence and evolution of AMR and, in the long term, support development of urgently-needed intervention strategies.

## Supplementary material

Supplementary File 1: A fully formatted report with all Tables and Figures and Appendices entitled “AMR Surveillance in low- and middle-income settings. A Roadmap for Participation in the Global Antimicrobial Surveillance System (GLASS).”
