# Supplementary material for: AMR Surveillance in low and middle-income settings - A roadmap for participation in the Global Antimicrobial Surveillance System (GLASS)
Source: Wellcome Open Res. 2017 Sep 26;2:92. [Version 1] doi: 10.12688/wellcomeopenres.12527.1 (PMC5645727; doi:10.12688/wellcomeopenres.12527.1)
Supplement: Supplementary file 1 [file wellcomeopenres-2-13565-s0000.tgz › 99f63366-743d-473c-b3ad-e96403e4ab3e.pdf]

Funded by UK Government

2016

Fleming Fund

AMR Surveillance in low- and middle-income settings

A ROADMAP FOR PARTICIPATION IN THE GLOBAL ANTIMICROBIAL SURVEILLANCE SYSTEM (GLASS)

Contents

Contents 2
Abbreviations 3
Foreword 4
Executive Summary 5

1. Introduction 6
1.1 AMR in low-income countries 6
1.2 Development of the roadmap for AMR Surveillance implementation 6
1.3 Legal and ethical considerations 9

2. Steps to establishing AMR Surveillance 10
2.1 National Action Plan 10
2.2 Governance and structures 12
2.2.1 National Coordinating Centre 12
2.2.2 External organisations 13
2.2.3 Site coordinating committee 14
2.2.4 Laboratories 15
2.3 Situational analysis 16
2.4 Training 16
2.5 Sentinel site identification 16
2.6 Levels of AMR surveillance 17

3. Technical components for AMR surveillance 19
3.1 Overview 19
3.2 Population catchment and sampling frame 19
3.3 Clinical surveillance 19
3.4 Patient sampling 20
3.5 Isolate identification 20
3.6 Antimicrobial susceptibility testing 21
3.7 Data management 22
3.8 Use of innovative technologies and mobile communications 23

4. Monitoring, evaluation and development 25
4.1 Quality assurance 25
4.2 Clinical QA 25
4.3 Laboratory QA 25
4.4 Data systems QA 26
4.5 Key Performance Indicators 26
4.6 Development of AMR surveillance platforms 27
4.6.1 Assessing antimicrobial usage 27
4.6.2 Research 27

5. References 28

6. Acknowledgements 29

7. Appendices 30
Appendix A: Key documents 30
Appendix B: Clinical assessment of adults - syndromic diagnoses 31
Appendix C: Clinical assessment of children - syndromic diagnoses 33
Appendix D: National functions for AMR Surveillance 34
Appendix E: Sentinel site functions for AMR Surveillance 36
Appendix F: Minimum level of identification required for the eight priority pathogens 38
Appendix G: Pathogen-antimicrobial combinations 38

LONDON SCHOOL of HYGIENE & TROPICAL MEDICINE

Abbreviations

**AMR**	Antimicrobial Resistance
**AMRS**	Antimicrobial Resistance Surveillance
**AST**	Antimicrobial Susceptibility Testing
**CAESAR**	Central Asian and Eastern European Surveillance of AMR
**CDC**	US Centers for Disease Control and Prevention
**CLSI**	Clinical and Laboratory Standards Institute
**CSF**	Cerebrospinal fluid
**DfID**	Department for International Development
**DH**	Department of Health
**DRI**	Drug Resistant Infection
**EQA**	External Quality Assurance
**EARS-Net**	European AMR Surveillance Network
**EUCAST**	European Committee on Antimicrobial Susceptibility Testing
**FAO**	Food and Agriculture Organization
**FTP**	File Transfer Protocol
**GHS**	Global Health Security
**GLASS**	Global AMR Surveillance System
**HDSS**	Health and Demographic Surveillance System
**IHME**	Institute of Health Metrics Evaluation
**IQA**	Internal Quality Assurance
**KPI**	Key Performance Indicator
**LMIC**	Low and Middle Income Countries
**MIC**	Minimum Inhibitory Concentration
**NAP**	National Action Plan
**NCC**	National Coordinating Centre
**NRL**	National Reference Laboratory or coordinating AMR laboratory
**PHE**	Public Health England
**QA**	Quality Assurance
**QC**	Quality Control
**ReLAVRA**	Latin American AMR Surveillance Network
**SCC**	Site Coordinating Committee
**SOP**	Standard Operating Procedures
**STI**	Sexually Transmitted Infection
**UKNEQAS**	United Kingdom National External Quality Assurance Scheme
**WHO**	World Health Organization
**WHONET**	World Health Organization laboratory database software

Foreword

Antimicrobial resistance poses a catastrophic threat on a global scale. Drug resistant infections are already on the rise with numbers suggesting that up to 50,000 lives are lost each year to antibiotic-resistant infections in Europe and the US alone. Globally, at least 700,000 die each year of drug resistance in illnesses such as bacterial infections, malaria, HIV/AIDS or tuberculosis.

The UK is at the forefront of the global fight against antimicrobial resistance, commissioning an independent analysis of this global problem and proposing concrete actions to tackle it internationally, and now with the £265 million Fleming Fund working to strengthen surveillance of drug resistance and laboratory capacity in developing countries, the AMR Innovation Fund and the implementation of the UK AMR Strategy 2013–18.

Globally, the threat of AMR has been recognised by the adoption of a Global Action Plan on antimicrobial resistance at the World Health Assembly, by resolution 4/2015 adopted at the Food and Agriculture Organization's Conference and Resolution No. 26 adopted at the General Session of the World Assembly of National Delegates to the World Organisation for Animal Health; all in 2015. All countries have committed to the implementation of national action plans to address AMR. These plans need to include surveillance systems and capacity to improve diagnosis of infectious diseases, monitoring of the prevalence of resistance, and monitor antibiotic prescription and use.

A key challenge in meeting the requirements of the Global Action Plan is the lack of surveillance data on resistance and antimicrobial prescription and use. This is particularly acute for many developing countries where there is the expectation that drug-resistant infections will have a disproportionate impact. This is why, taking a One Health approach, the UK's Fleming Fund will support improvements in data and surveillance of antimicrobial resistance in Sub-Saharan Africa, South and Southeast Asia.

I strongly welcome the development of this protocol which I hope will support countries in developing comprehensive and quality assured surveillance systems so that they are able to implement the World Health Organization's Global AMR Surveillance System (GLASS). This is one of a number of measures designed to tackle the problem of AMR.

While the focus of the Fleming Fund is specific regions where this will be tested, it has been designed for use in all countries.

The protocol addresses a real need and I very much welcome its publication.

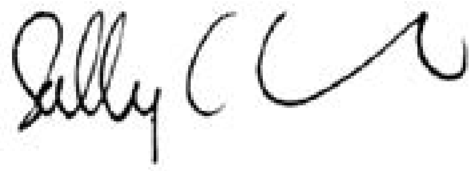

Chief Medical Officer, England

Executive summary

Drug-resistant infections, caused by bacteria with increasing antimicrobial resistance (AMR), threaten our ability to treat life-threatening conditions. Tackling AMR requires international collaboration and partnership. An early and leading priority to do this is to strengthen AMR surveillance, particularly in low- and middle-income countries where the burden of infectious diseases is highest and where data are most limited.

The World Health Organization has developed the Global AMR Surveillance System (GLASS) as one of a number of measures designed to tackle the problem of AMR, and WHO member states have been encouraged to produce National Action Plans for AMR by 2017. However, low- and middle-income countries are unlikely to have the resources or capacity to implement all the components in the GLASS manual. To resolve this, we have developed a guideline that is aligned to the GLASS procedures, but written specifically for implementation in low- and middle-income countries. The guideline allows for flexibility across different systems, but has sufficient standardisation of core protocols to ensure that, if followed, data will be valid and comparable. This will ensure that the surveillance programme can provide health intelligence data to inform evidence-based interventions at local, national and international levels.

1 Introduction

1.1 AMR in low-income countries

AMR develops when strains of micro-organisms evolve to survive exposure to antimicrobial agents. The subsequent transmission and spread of resistant pathogenic bacteria results in drug-resistant infections (DRIs). The increasing use of antimicrobials worldwide has been associated with a global increase in DRIs, which threatens to return clinical therapies to the pre-antibiotic era. At present, DRIs are estimated to account for 50,000 deaths each year in Europe and the USA alone,[1] but by 2050 it is estimated that DRIs will account for 10 million deaths per year worldwide; posing an economic and biosecurity threat.[2]

Countries with the highest burdens of communicable diseases usually have the least resources and, in these settings, data on AMR and DRIs are most limited.[3,4] While large regional AMR surveillance networks have been established in Europe (EARS-Net), Latin America (Red Latinoamericana de Vigilancia de la Resistencia a los Antimicrobianos, ReLAVRA) and Central Asia and Eastern Europe (CAESAR), capacity for AMR surveillance in low- and middle-income countries (LMICs) is relatively limited and fragmented, despite evidence that, as with the rest of the world, AMR in LMICs is increasing.[3]

The importance of strengthening AMR surveillance in LMICs was highlighted in 2014 by a United Kingdom government-commissioned review.[1] In response, the United Kingdom Department of Health (DH) launched the Fleming Fund to support LMICs in developing AMR surveillance systems. The fund is aligned with the World Health Organization's Global AMR Surveillance System (GLASS)[5] to support the Global Action Plan on AMR.[3]

The aims of the WHO AMR surveillance programme include monitoring trends in infection and resistance to develop standard treatment guidelines that support best practice for patient care, but also recognise the importance of linking information on AMR from different sectors, such as human, animal, food, agriculture, environment, and data on antibiotic use in human and animal populations and environmental antibiotic usage. AMR surveillance should also allow for assessment of interventions to reduce AMR, provide early alerts for emergence of novel resistant strains, and aid the rapid identification and control of outbreaks.[6]

1.2 Development of the roadmap for AMR surveillance implementation

The aim of this work is to facilitate AMR surveillance and participation in GLASS for LMICs. Recognising that capacity varies considerably, we describe an approach that allows the independent development of each component of surveillance to build a comprehensive system. We recommend a sentinel surveillance system (see key surveillance definitions)[7] with step-wise expansion to increase the numbers of and scope of participating sites. In the first instance we propose that countries should identify or develop capacity in a single site that can undertake surveillance to an acceptable core standard. Having achieved that standard, the primary site should support the development of good practice in secondary sites, with the long-term aim of building a comprehensive network of sentinel sites which can

provide high-quality representative AMR data. Sentinel sites that have achieved core capacity may aspire to higher standards (extended and advanced, Appendix D) by developing and extending their capabilities.

This guideline has been developed with the objective of supporting capacity development in a standardised manner while allowing flexibility to incorporate country or regional priorities. It is intended to:

- be suitable for use by LMICs, recognising the context of different health systems;
- be based on an assessment of available evidence and review of established protocols in comparable resource settings;
- provide a basis for early collection and analysis of data on AMR that will help countries to assess the extent of AMR in important pathogens and participate in global and regional surveillance (GLASS);
- take into account the need for epidemiological and statistical validity and quality assurance, so that the data can be used, shared and combined to provide reliable evidence of AMR prevalence and to evaluate the effectiveness of interventions;
- provide a tiered structure, with a minimum level of essential (core) activities and scope for expansion so that countries can select the level of operation to suit their circumstances, with the ability to expand and broaden to advanced surveillance activities over time;
- provide a roadmap for improving laboratory capacity, data collection and surveillance for AMR with an effective One Health approach, through multi-sectoral involvement across the interface between humans, animals and their various environments.

While recognising the global importance of drug resistance among viruses, fungi and parasites, this document focuses on bacterial infections in humans, and particularly on eight pathogens identified by the WHO as priority organisms for the early implementation of AMR surveillance. However, we anticipate that activities which improve the isolation, identification, susceptibility testing and reporting of these organisms will support development of clinical diagnostics for other pathogens, and can be tailored in-country for locally important or emergent bacteria.

Similarly, while the emphasis in this guideline is on human clinical pathogens we recommend, in line with WHO, that AMR surveillance, in the long term, be embedded in a One Health approach. In this context, it is expected that AMR surveillance systems will develop in LMICs progressively to include agriculture (including animal health) and the environment. These activities are not included here because they are normally conducted by a parallel laboratory system, but they should be considered as the capacity for AMR surveillance in clinical settings advances. To support this, there should be multi-sector representation (including involvement from agriculture and veterinary medicine) in AMR surveillance bodies from the outset, in order to inform, monitor and control the threat to public health arising from AMR.

Key surveillance definitions

Public health surveillance: Public health surveillance is the continuous, systematic collection, analysis and interpretation of health-related data needed for the planning, implementation, and evaluation of public health practice (WHO).

Active surveillance: staff members contact heath health care providers or the population to seek information about health conditions.

Passive surveillance: staff members receive routine reports from health care providers.

Continuous surveillance: ongoing surveillance not limited to specific time periods.

Episodic surveillance: repeated episodes of surveillance for defined periods of time.

Enhanced surveillance: collection of specific data related to the target disease, in addition to routine data collection.

Comprehensive surveillance: includes all health care providers and/or laboratories in the surveillance system to report all cases of a defined condition.

Sentinel surveillance: a prearranged sample of representative health-care providers and/or laboratories agrees to report all cases of defined conditions, which might indicate trends in the population as a whole.

Population-based surveys: use of standardised questionnaires, investigations and protocols to assess population levels of particular health conditions and/or other characteristics, such as the Demographic and Health Surveys undertaken regularly in low-income countries.

Laboratory-based surveillance: can include syndromic surveillance, laboratory-confirmed surveillance and integrated surveillance depending on in-country capacity.

Integrated Disease Surveillance and Response: links epidemiologic and laboratory data in communicable disease surveillance systems across health facilities with associated public health action.

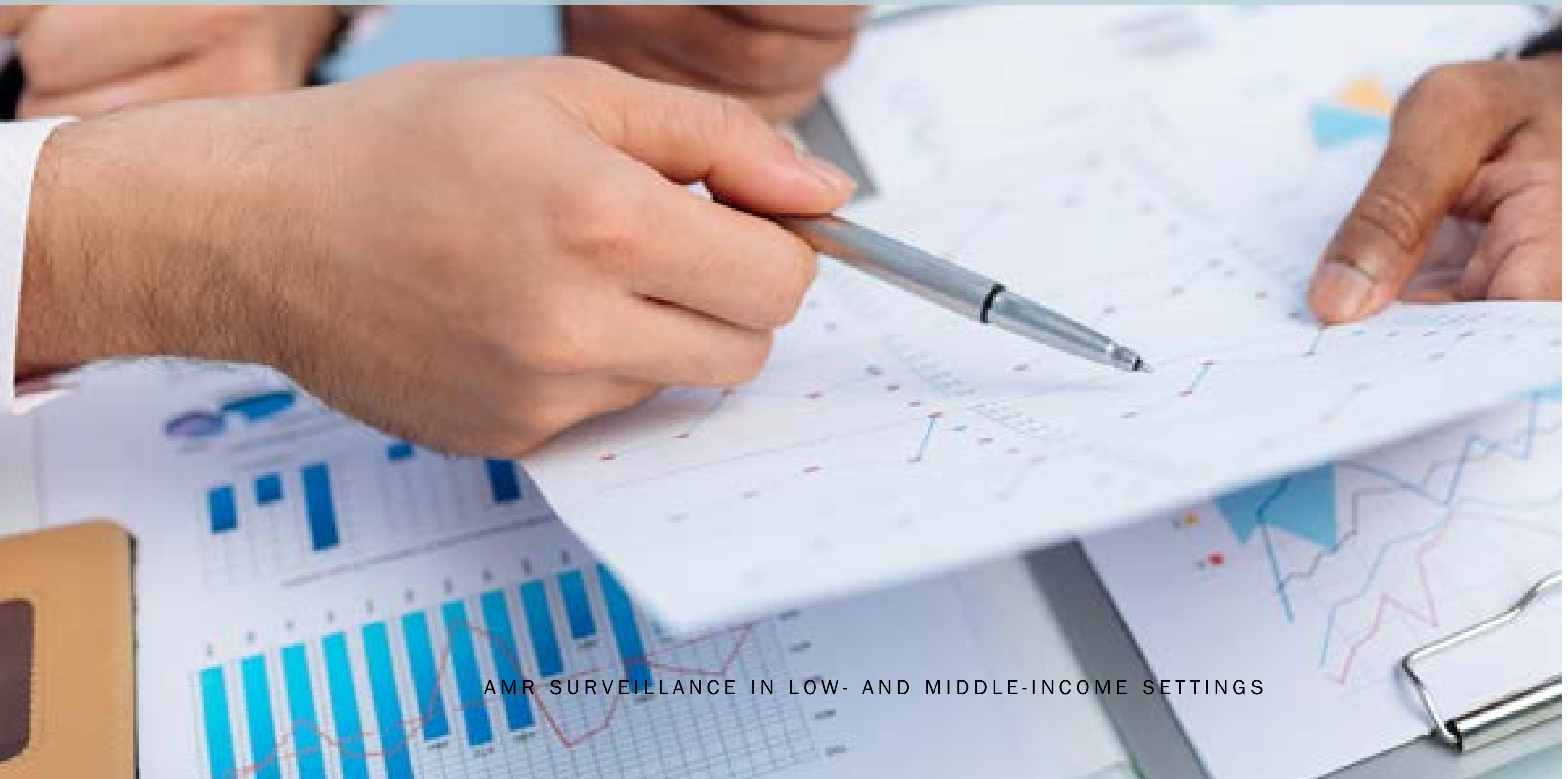

1.3 Legal and ethical considerations

Public health surveillance is usually legally mandated by the national government. For public health surveillance programmes, the probability and the magnitude of harm to the population arising from not reporting surveillance data must be moderate to major to justify the use of individual patient data without individual patient consent.[8] In this context, the WHO has recently recognised AMR as a significant potential global health threat.[9] Reporting the characteristics of resistant pathogens rarely represents a threat to patient confidentiality, however, the inclusion of simple clinical data such as age, sex, collection date, specimen type and syndromic diagnosis, adds considerable value to the information obtained from the laboratory, and there are clear benefits from AMR surveillance at patient, pathogen and population levels.[6]

Examples of the application of AMR data include:

- timely feedback to clinicians to support patient care and enable rationalisation of antibiotic treatment; use of data to inform local antimicrobial prescribing guidelines and infection control policies;
- analysis of clinical surveillance data (at international, national and/or local level) to enable public health interventions;
- cross-policy collaboration and support for research institutions to analyse clinical surveillance data, adopting a One Health to understand the emergence, transmission and dissemination of pathogens at the human-animal interface.

Given the need to integrate data from different sources, including individual patient data, it is essential that there are data governance agreements and procedures in place. These should protect the confidentiality of individual patients but also facilitate the sharing of AMR surveillance data to inform policy locally, nationally and internationally. To meet ethical obligations, technical, legal and/or political barriers to data sharing[10] must be overcome, and best practice for data collection ensured (see section 3.7). For these reasons, a successful AMR surveillance programme requires clear political support, and should engage accordingly with the relevant government bodies.[10]

2 Steps to establishing AMR Surveillance

2.1 National Action Plan

The first step in establishing AMR surveillance is the development of a National Action Plan (NAP) for AMR (figure 1), as set out by the Global Action Plan on AMR.[3] WHO member states have been encouraged to develop NAPs for AMR by 2017 and a manual and template are available to support this process (Appendix A). Some countries are already participating in this process, as described in the case study for Vietnam.

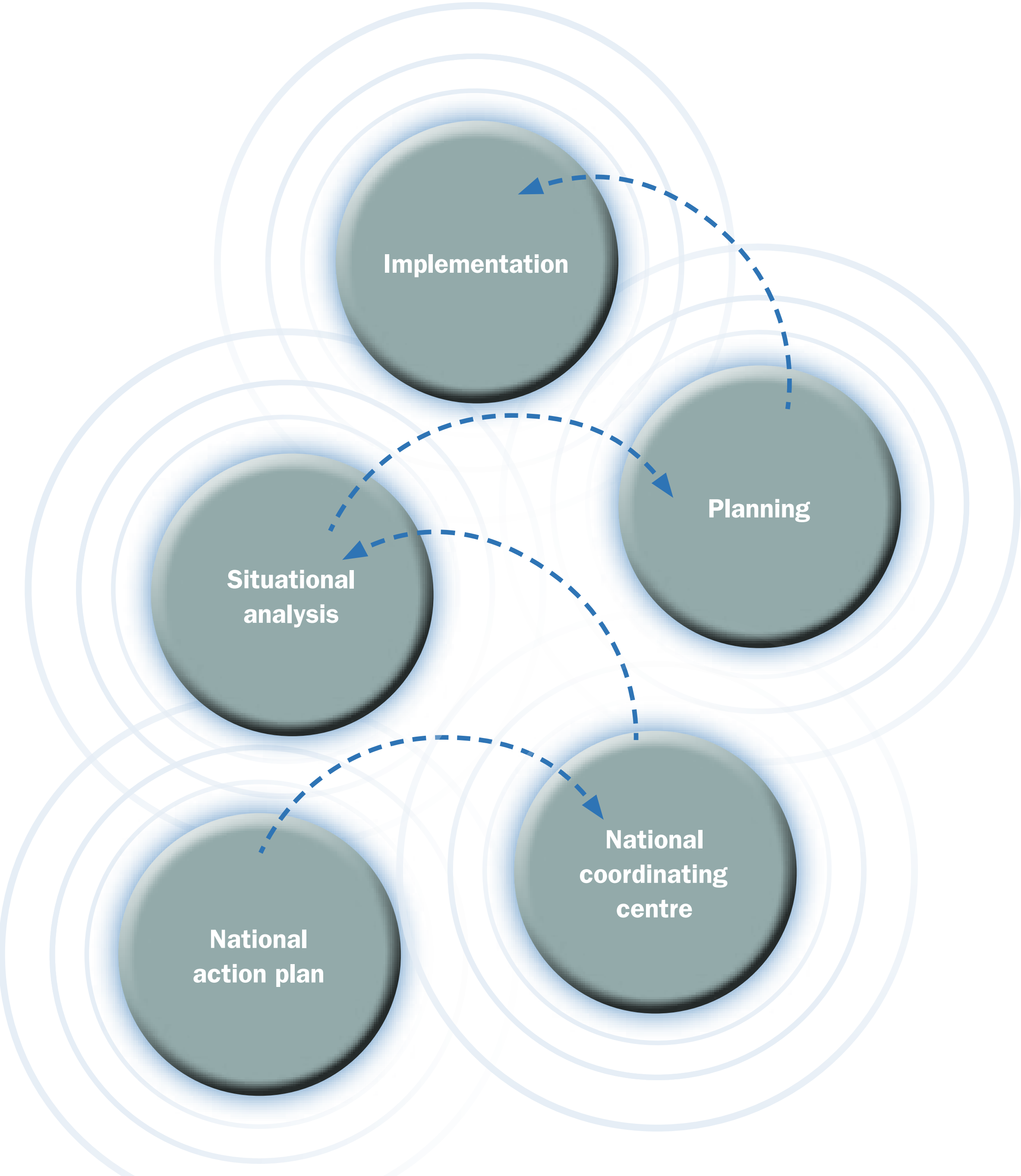

Figure 1: Steps in starting up AMR surveillance

Case study - Vietnam

Vietnam was the first country in the World Health Organization’s Western Pacific Region to approve a National Action Plan (NAP) in 2013 to combat drug resistance.

On 22 July 2015, the Ministry of Health, the Ministry of Agriculture and Rural Development, the Ministry of Trade and Industry and the Ministry of Natural Resources and Environment (MONRE) signed an aide-memoire to coordinate and jointly implement the NAP for AMR across different sectors.

The aide-memoire and NAP also help to raise awareness about AMR, support capacity of national surveillance systems on antibiotic use and resistance, ensure adequate supply of quality essential drugs and strengthen safe and rational drug use and infection control across sectors.

Situational analyses have followed on antibiotic use and resistance in Vietnam, including examination of the healthcare system, drug regulation and supply, antibiotic resistance and infection control, and agricultural antibiotic use. These were done by reviewing international and local reports as well as through discussions with stakeholders.

Subsequent to this, Vietnam is developing a new AMR reference laboratory in Hanoi, supported by the UK’s Fleming Fund, and is establishing a network of sentinel surveillance sites across the country.

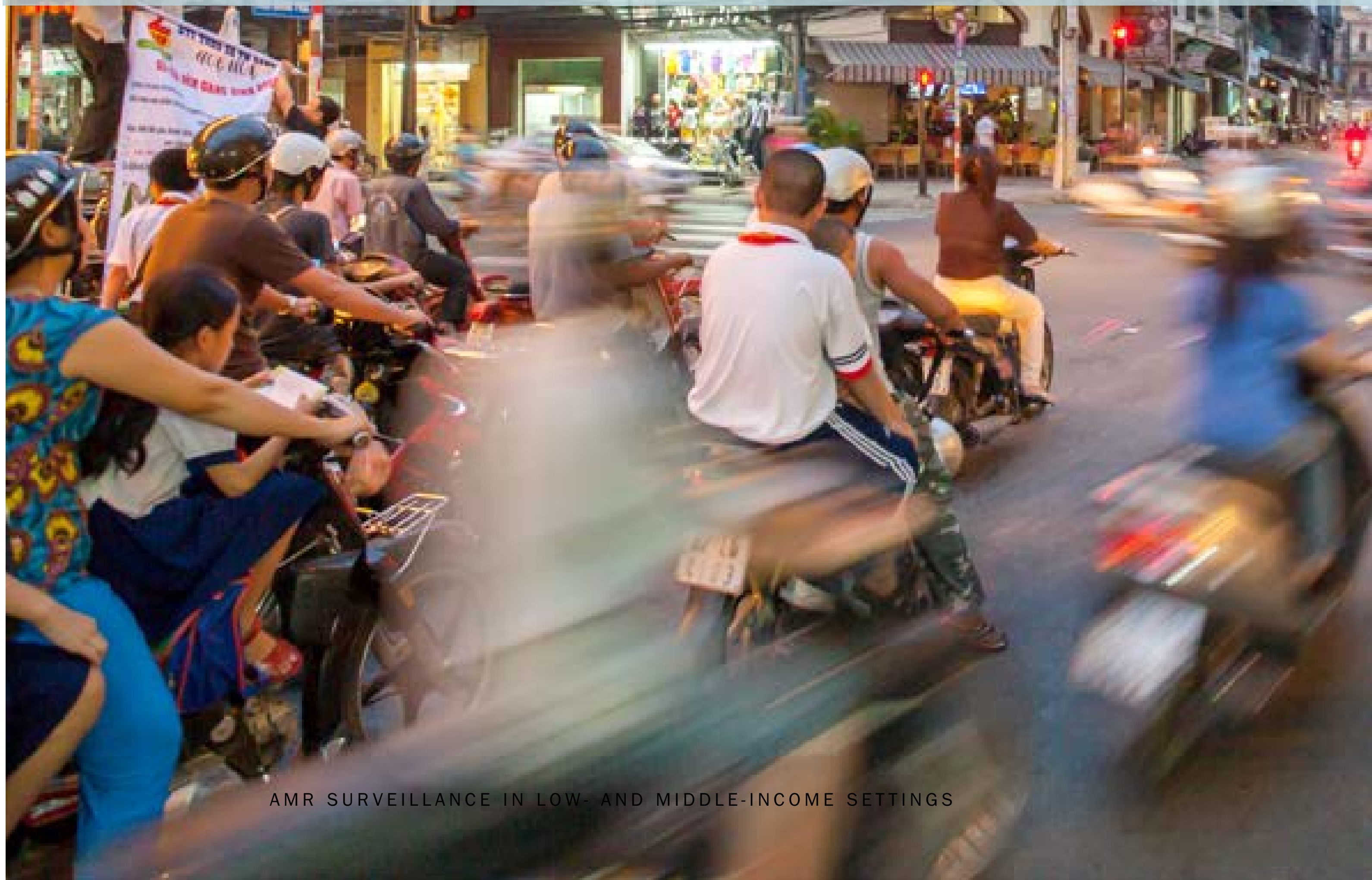

2.2 Governance and structures

Each country should develop its own organisational structures (figure 2), and define terms of reference. While the governance structure may vary, important factors include identification of a National Coordinating Centre (NCC), convening a technical team, and strong engagement with the Ministry of Health, reflecting the national importance of AMR surveillance in health systems.

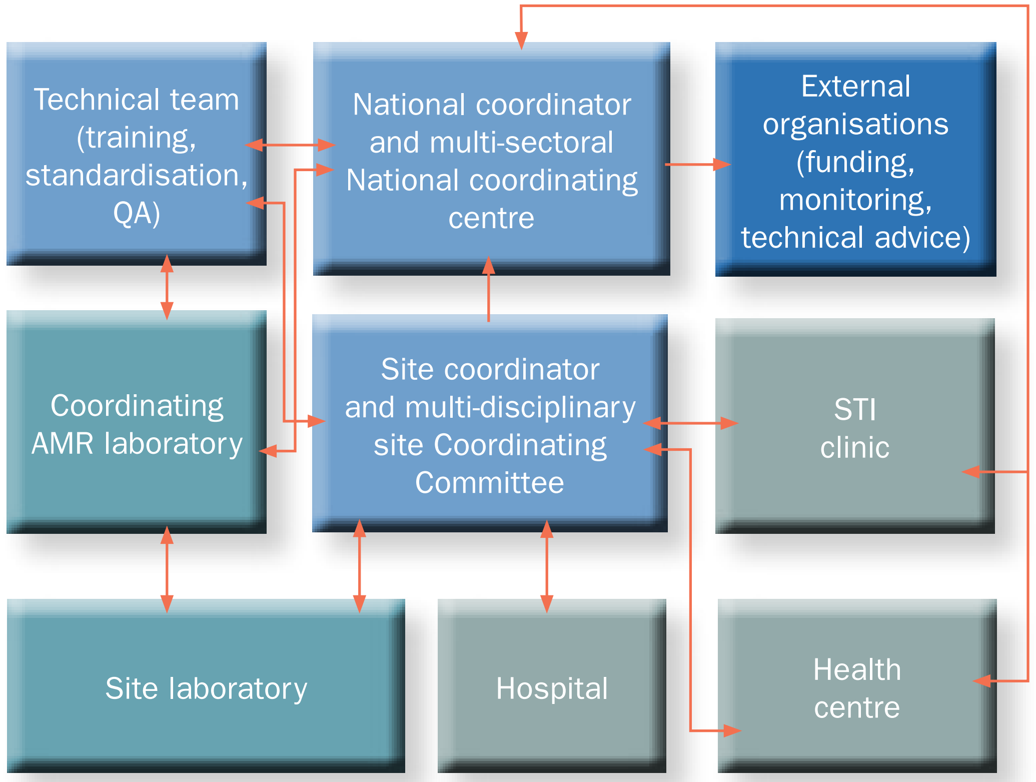

Figure 2: Example organisational structure for AMR surveillance in low resource settings

2.2.1 National Coordinating Centre

The NCC should include a committee of multi-sectoral stakeholders to support a One Health approach at national and international levels. This committee could be developed from the national working group on AMR, as established by the Global Antimicrobial Resistance Partnership (GARP), or the committee responsible for the NAP. The committee should report to the appropriate national body, for example the Ministry of Health, and, where appropriate, collaborate with a relevant external organisation/funder.

The roles and responsibilities of the committee should be set out with formal terms of reference. Membership should include relevant technical experts and stakeholders although individuals may fulfil the remit of more than one technical brief. A typical committee may include a variety of roles and representatives of a range of institutions, as below:

- technical team leader
- Ministry of Health
- Ministry of Agriculture
- national public health institute

- coordinating AMR laboratory
- international stakeholders
- clinical microbiologist
- data manager
- public health analyst
- laboratory manager
- hospital manager
- adult physician
- paediatrician
- pharmacist
- veterinarian
- infection control manager

The functions of the national coordinating centre include:

- commissioning a situational analysis of current capacity and sustainability for AMR surveillance
- national strategic planning for AMR surveillance
- oversight of AMR surveillance implementation at a national level against key performance indicators

The strategic function may be extended to include other aspects of tackling AMR, for example strategic oversight of infection prevention and control (IPC) policy, development and use of standardised treatment guidelines, and control of the sale of antimicrobial agents.

The NCC has oversight of the technical team whose responsibilities are to:

- monitor quality assurance
- support capacity building through training of national and site level participants
- determine national priorities for pathogens in AMR surveillance in addition to those identified as priority pathogens by the WHO
- review the scope of AMR surveillance as capacity develops, and to integrate a One Health approach
- review the introduction of new technologies
- support research programmes to use AMR surveillance platforms
- collaborate with neighbouring countries and across international regions
- develop and expand regional networks

The NCC is headed by a named National Coordinator for AMR surveillance from a key stakeholder institution such as the Ministry of Health, Institute of Public Health, or similar organisation. The National Coordinator is supported by a technical team responsible for training, standardisation and quality assurance. Where appropriate, the technical team may be led by the National Coordinator.

2.2.2 External organisations

The NCC will collaborate with international stakeholders and funding bodies such as the Fleming Fund, the US Centers for Disease Control and Prevention, the Institut Pasteur, the European Centre for Disease Prevention and Control, the Bill & Melinda Gates Foundation, and major non-governmental organizations including Médecins sans Frontières, the Global Health Security Agenda, the Food and Agriculture Organization, the World Organisation for Animal Health, and the World Health Organization.

The NCC should work with external bodies to ensure standardisation, training and internal

and external quality assurance (QA) of all processes relating to AMR surveillance across participating countries, for example by developing and participating in national and international workshops.

2.2.3 Site coordinating committee

Sentinel sites should determine and define their own organizational structures, and how this fits into existing hospital and laboratory administration systems. There should, however, be a Site Coordinating Committee (SCC), with defined terms of reference, and which includes relevant representatives, for example:

- site leader
- hospital administrator
- data manager
- laboratory manager
- clinical microbiologist
- adult physician
- paediatrician
- infection control manager
- pharmacist
- veterinary practitioner
- public health specialist

The site leader would be expected to have project management and programme implementation skills, and should report to the NCC.

The role of the SCC, led by the site coordinator, includes:

- working with the national technical team to facilitate a situational analysis of current capacity and sustainability at the site
- planning strategic priorities at the site
- oversight of AMR surveillance implementation at the site and reporting against key indicators

The roles of the SCC are to:

- support on-site training for AMR procedures
- develop locally-adapted standard operating procedures (SOPs)
- ensure quality control measures and regular audit for all AMR surveillance processes
- work with the national technical team to establish internal quality assurance assessment, progressing to external quality assurance assessment
- ensure effective lines of communication are in place for feedback of AMR results to clinicians and feedback of summarised AMR data to local participants and stakeholders (administration, clinical, laboratory and data staff)
- report anonymised case-level data to the National Coordinator in a timely manner

The strategic function of the SCC may be extended to include other aspects of tackling AMR, for example, ensuring nationally agreed infection prevention and control policies and treatment guidelines are being followed.

2.2.4 Laboratories

A coordinating AMR laboratory should be established for AMR surveillance. This may already be in place, or may be developed as part of the capacity-building process. Where there is no capacity for a coordinating AMR laboratory, countries should collaborate with neighbouring countries, which may be able to provide this service.

Coordinating AMR laboratories should be accredited, or be working towards laboratory accreditation[11]. Their staff should be trained by the technical team and / or external partners to provide training for sentinel site laboratory staff, using a “Train the Trainers” approach (Appendix A). The functions of the coordinating AMR laboratory are:

- core laboratory processes as described in Appendix D
- participation in internal quality assurance
- participation in external quality assurance through appropriate international schemes
- provision of a reference service for core organism/antimicrobial combinations as a minimum, for borderline isolates or isolates with unexpected or unusual resistance profiles, and collaboration with international centres to monitor emerging resistance patterns
- assisting sentinel site laboratories to procure equipment and reagents, in collaboration with the NCC
- maintaining a biorepository for bacterial isolates
- promotion of good practice (including development of national SOPs) to ensure standardisation and quality control
- training staff at sentinel site laboratories
- facilitating the development of internal quality assurance at sentinel site laboratories
- provision of external quality assurance across sentinel site laboratories if they do not already participate in EQA (for example, by testing a subset of isolates from the sentinel site laboratories and providing feedback)

Each sentinel site should have its own laboratory, or access to a laboratory, which is able to:

- provide core laboratory processes, including isolate identification, susceptibility testing and storage as described in Appendix E
- communicate AMR results (organism and susceptibilities) to clinicians in a timely manner to improve the care of individual patients
- refer isolates with unusual, unexpected or indeterminate resistance patterns to the coordinating AMR laboratory for further testing
- participate in on-site training and attend national training as appropriate
- adhere to localised SOPs with quality control checks
- conduct internal quality assurance procedures
- work with the technical team and coordinating AMR laboratory to develop capacity, working towards participation in EQA and gaining laboratory accreditation

2.3 Situational analysis

A situational analysis of AMR should be undertaken nationally. This should consider all aspects of AMR surveillance, including clinical sampling, laboratory procedures and data systems. A detailed laboratory assessment can be performed using the World Health Organization's Laboratory Assessment Tool (Appendix A).

2.4 Training

To promote awareness of AMR surveillance, education and training should be integrated into local and national education programmes, across all the disciplines required for AMR surveillance. These include clinical, laboratory, information technology and public health training (figure 3). Teaching on AMR should be introduced into formal training pathways, including undergraduate and postgraduate curricula across these disciplines. AMR awareness should also be developed through continuing professional development (training days, workshops) at site, regional, national, and international levels. Such training should incorporate e-learning options and specific training modules. To enhance motivation, site coordinating committees should consider appointing individuals with specific roles to act as AMR surveillance champions in clinical (including infection prevention and control), laboratory and data services.

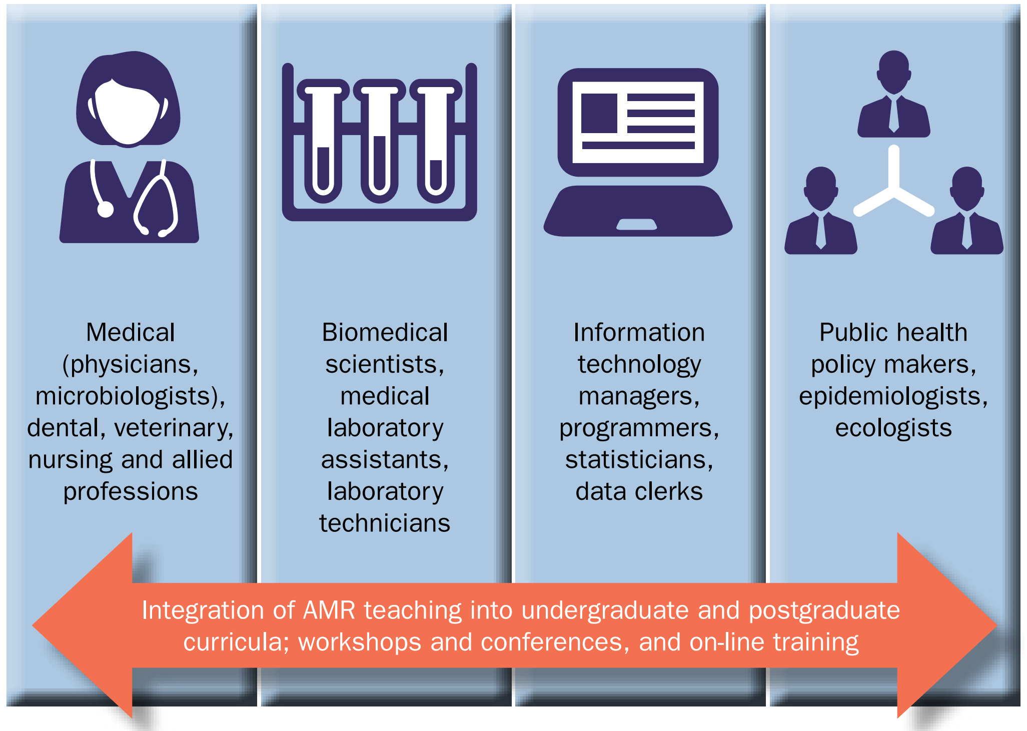

Figure 3: Integration of AMR surveillance into training and education

2.5 Sentinel site identification

The initial situational analysis should identify potential sites for AMR sentinel site surveillance. Site selection should be undertaken by the NCC through a transparent process, with involvement of an external stakeholder or funder where appropriate.

The sites selected, and the network as a whole, should reflect relevant variations in geography, socioeconomic factors and demography, disease epidemiology (e.g. co-morbidities such as HIV) and ecology, taking into account climate, rainfall and land use.

Surveillance that only represents one level of healthcare (e.g. referral hospitals) will not adequately reflect the AMR situation of the country. The potential for biases include:

- sampling only from referral hospitals, which may have high numbers of patients treated with antibiotics prior to sampling or high numbers of cases who have failed first-line treatment at referring facilities
- sampling only from hospitals may under-represent less severe infections e.g. sexually-transmitted infections, uncomplicated urinary tract infections, community acquired pneumonia.
- sampling only from healthcare outpatient clinics will result in under-representation of severe or invasive infection
- health financing systems that require patients to pay for investigations will include only those who are able to afford investigations

AMR surveillance sampling should therefore be drawn from the health facilities used by the population targeted for surveillance. These may include referral hospitals, district hospitals and out-patient facilities (including primary care); some institutions may fulfil more than one of these functions. Facilities serving a population sub-group, such as private hospitals in a country where most hospital services are delivered through the public sector, should only be included to the extent that the private sector supplies care in that country.

It is anticipated that sites and settings will be identified with very different levels of capacity (see Box 1). At the initiation of AMR surveillance it is important to identify organisational and leadership strengths in order to develop systems and technical capacity. Key factors to consider when evaluating the potential of individual sentinel sites are:

- whether the site has capacity and support (from local management/government/ populations) to connect to a district or national network and subsequently share data with international agents including the WHO
- whether the site will be able to contribute to the national network through mentoring and supporting capacity building at subsequent sites
- what level of investment will be required to achieve and sustain core AMR surveillance participation

Once a site has been identified as a potential AMR sentinel surveillance site, a more detailed technical analysis should be performed to determine which **core/extended/advanced (Appendix E)** activities are being performed to the required standards, and what investments are required to facilitate full participation in surveillance.

2.6 Levels of AMR surveillance

To reflect variation in capacity between countries and regions, **core**, **extended** and **advanced** functions of AMR surveillance are described here, with the aim of prioritising a **core** standard to ensure basic quality data (appendices D and E). When these **core** processes are functioning at acceptable standards, sentinel sites should consider extending their capacity to include

extended, and/or **advanced** functions, and to support other sites to develop their capacity.

The choice of target level of surveillance should depend on:

- Current in-country capacity in clinical, laboratory and data handling areas
- Start-up and ongoing costs of the proposed AMR surveillance system
- Sustainability of the proposed AMR surveillance system

Box 1: Examples of country scenarios

High quality clinical and microbiological data available but not currently integrated into surveillance systems

This scenario describes a country where there are a number of broadly representative hospitals with good standards of clinical examination and recording using standard diagnostics, a functioning microbiology laboratory and the physical, legal and ethical capacity to link laboratory and clinical records for anonymised aggregation at individual case level, but without national oversight for AMR surveillance.

One or a few hospitals with acceptable clinical and microbiological data

This scenario describes a country where there may be a small number of hospitals with adequate standards of clinical examination and a functioning microbiology laboratory processing reasonable numbers of specimens for culture. Record keeping may not be formalized or electronic, but there is sufficient communication between the ward and the laboratory to link laboratory data to patients. Institutions may be government run, mission hospitals, or privately operated.

Research 'Centre of Excellence'

This scenario applies to a country with minimal public hospital resources to do AMR surveillance but where there is an academic centre of excellence undertaking clinical and / or microbiological surveillance.

No obvious facilities in country

This applies where there are no hospitals or research centres currently conducting clinical care and microbiological investigation to a standard sufficient for AMR surveillance.

Technical components for AMR surveillance

3

3.1 Overview

To allow full and informative interpretation of data, effective AMR surveillance requires well-functioning health-systems that serve a defined population. Standard laboratory methods for pathogen identification and antimicrobial susceptibility testing are vital in order to understand the emergence of AMR and inform policy, but so too are population descriptors, healthcare utilisation patterns, and the systematic assessment and investigation of patients (figure 4).

3.2 Population catchment and sampling frame

Wherever possible, the catchment population of the health facilities included in surveillance should be defined and an assessment should be made of the patterns of healthcare utilisation in that population. This is important for data interpretation: total population allows estimates of incidence and trends; descriptors define risk factors (socio-economic status, urbanisation, co-morbidity levels) for national models of AMR burden; access to care patterns determine whether the healthcare facilities included are the first point of contact, post-treatment, or post-clinical failure level – which will have different AMR prevalence. Health facility selection is an important part of sentinel site selection (see section 2.5) and a sentinel site laboratory should receive samples from both inpatient and outpatient clinic facilities, with costs associated with AMR surveillance covered at an institutional or national level, rather than directly charged to patients.

At the **extended** level, a healthcare utilisation survey would be appropriate, and at an **advanced** level the population catchment should be described using census data or by an enumeration survey. It may also be appropriate to make use of existing Health and Demographic Surveillance Systems (HDSS).[6]

3.3 Clinical surveillance

AMR surveillance data should be interpreted in the context of local clinical practice. This is particularly relevant for low- and middle-income country settings which use syndromic management approaches where patients are diagnosed clinically and treated empirically.

At a **core** level, the clinical data on the laboratory request form should include the clinical diagnosis selected from a list of syndromes. For adults this includes sepsis, severe pneumonia, acute diarrhoea, bacterial meningitis, severe soft tissue infection, pyelonephritis, sexually transmitted infections (Appendix B) or other (to allow for clinical discretion). The clinical syndromes for children include severe diarrhoeal disease, severe febrile illness, meningitis, severe pneumonia and, in neonates, possible serious bacterial infection (Appendix C).

At an **extended** level, clinical assessment of adults and children (<5 years) should be based on standardised and systematic history and examination with case definitions from national and international guidance (suggested in appendices B and C).[12] At an **advanced** level diagnosis would be supported by clinical proformas with electronic storage of these extended clinical data (to be electronically linked to laboratory data).

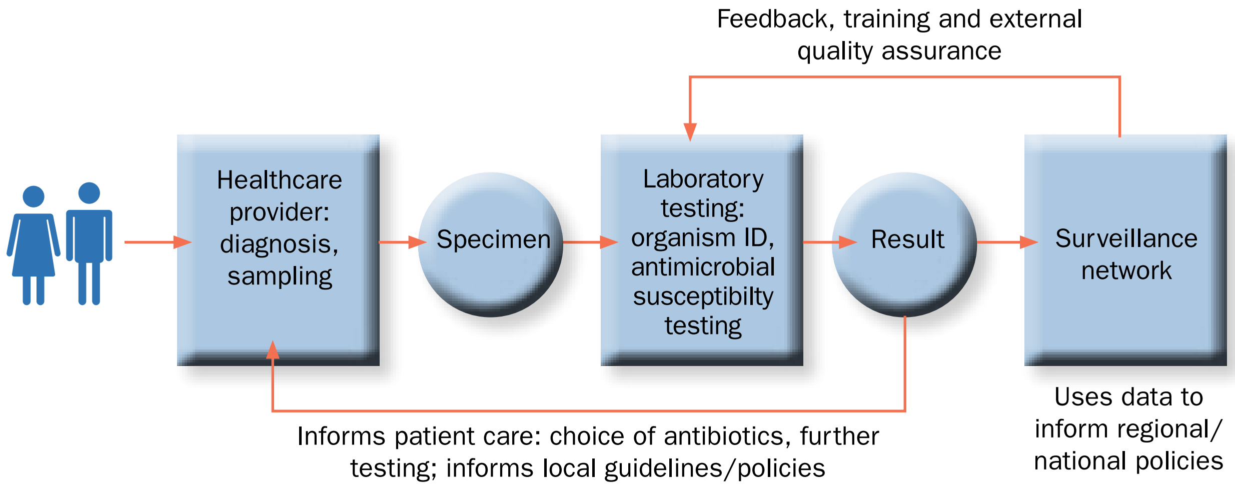

Figure 4: AMR Surveillance Process[i]

3.4 Patient sampling

Clinical sampling for AMR surveillance should be guided by the syndromic diagnosis for which the patient is being treated, (see appendices B and C for suggested outline) with additional investigations undertaken at the clinician's discretion. This supports interpretation of the data to guide empiric therapies and reduces potential bias which may occur if only clinical treatment failures or the most seriously ill patients are investigated.

The core investigation for AMR surveillance is blood culture, which is a specific indicator of pathogens causing invasive and life-threatening disease. It is anticipated that sentinel site laboratories will also process other samples, however, capacity building and data collection should initially focus on blood cultures as a **core** function. Once blood cultures are collected and processed to an acceptable standard, the laboratory should be encouraged to focus on cerebrospinal fluid (CSF) as the next priority sample associated with serious disease. At the **extended** level, laboratories should also have capacity to process urine, stool and urethral/ cervical swabs to AMR surveillance standards.

Appropriate staff training and SOPs should be in place for all procedures including collection, transport, registration, processing and reporting of samples. Personal protective equipment should be available, and sample transport should be undertaken safely, securely and in a timely fashion (Appendix A for safety manuals and guidance documents).

3.5 Isolate identification

Specimen culture and testing for antimicrobial susceptibility should be done by sentinel site laboratories. Isolates with unusual susceptibility profiles, or of uncertain identification, should be referred to the coordinating AMR laboratory, as well as a proportion of all isolates for quality control purposes. All isolates cultured from blood or cerebrospinal fluid specimens should be sent to the coordinating AMR laboratory for storage.

[i]Adapted from. Crump JA, Youssef FG, Luby SP, et al. Estimating the incidence of typhoid fever and other febrile illnesses in developing countries. Emerging infectious diseases 2003; 9(5): 539-44.[13]

Reporting for AMR surveillance should focus on the eight WHO priority pathogens as described in the GLASS manual, and national priorities. These are

- *Escherichia coli*
- *Klebsiella pneumoniae*
- *Acinetobacter baumannii*
- *Staphylococcus aureus*
- *Streptococcus pneumoniae*
- *Salmonella* spp.
- *Shigella* spp.
- *Neisseria gonorrhoeae*

At **core** level, pathogens should be identified by using relevant biochemical and/or serological tests as described in Appendix F. At the advanced level, laboratories may use molecular methods and automated systems such as MALDI-TOF, Vitek or Microscan (Appendix E).

3.6 Antimicrobial susceptibility testing

AMR surveillance programmes should include at least the following bacteria-antimicrobial drug combinations in compliance with the GLASS manual (see Appendix G for all combinations):[1ii]

- *Escherichia coli* vs. 3rd generation cephalosporins and fluoroquinolones;
- *Klebsiella pneumoniae* vs. 3rd generation cephalosporins and carbapenems;
- *Staphylococcus aureus* vs. oxacillin or cefoxitin;
- *Streptococcus pneumoniae* vs. penicillin or oxacillin;
- *Salmonella* species vs. fluoroquinolones;
- *Shigella* species vs. fluoroquinolones;
- *Neisseria gonorrhoeae* vs. 3rd generation cephalosporins

Antimicrobial susceptibility testing for priority pathogens should be carried out in line with international standards, preferably according to the European Committee on Antimicrobial Susceptibility Testing (EUCAST) methodology and guidance (Appendix A). Where Clinical and Laboratory Standards Institute (CLSI) guidelines are used, these may also be reported. Unless automated systems are already in place, antimicrobial susceptibility testing at the **core** level should be performed using the disc diffusion method.

Where additional drugs are included (for example *Acinetobacter baumannii* vs. carbapenems), they should be tested according to accepted guidelines (e.g. CLSI, EUCAST).

Sentinel site laboratories should document whether isolates are susceptible, intermediate or resistant (S/I/R) according to clinical breakpoints defined by EUCAST or CLSI. Zone sizes (mm) should also be measured and recorded, to allow for retrospective adjustment if new breakpoints are set.

At the **extended** and **advanced** levels, minimum inhibitory concentrations (MICs) may be determined, e.g. by microbroth dilution (manual or automated) or gradient diffusion tests such as E-Tests. MIC values should be recorded (in case breakpoints change in the future).

[ii] Other combinations will be appropriate depending on the setting. For example consider adding macrolide susceptibility to S. *pneumoniae* testing where macrolides are widely used.

3.7 Data management

Reporting of results requires efficient data management at both sentinel site and national levels (figure 5). Quality control should be incorporated at every stage, with automated data validity checks and rules, as well as audit to check data consistency, completeness and accuracy. Confidentiality should be protected and data security measures should be in place (resources in Appendix A).

The site coordinator should ensure individual case-level anonymised data (as set out below) are submitted to the national coordinator with health facility data. These include the total number of patient episodes and the total number of samples processed in the laboratory. The site coordinator should feedback sentinel site data at least quarterly to healthcare administration, clinical and laboratory staff, to support continued engagement with AMR surveillance.

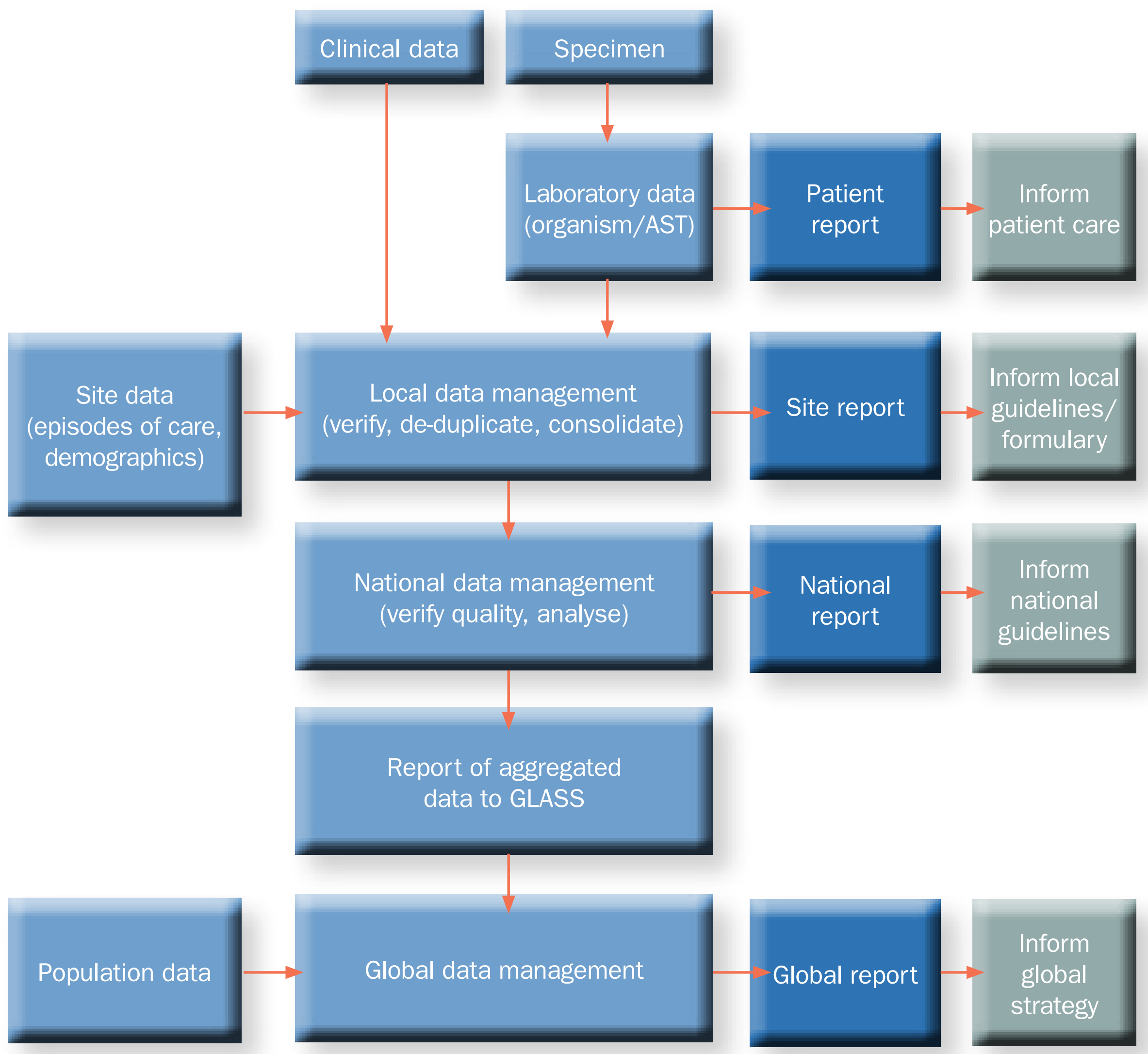

Figure 5: Data flows for AMR surveillance

Clinical data should be recorded in a standardised paper request form that accompanies the clinical sample to the laboratory for core surveillance. Sites operating at **extended** level will capture data using an electronic system. The minimum set of data required on the core clinical request form are: age, sex, clinical diagnosis, specimen type, sample date, admission date, hospital or community source.[14] Data fields collected at the extended level include: healthcare facility type (referral, district, health centre, general clinic, and STI clinic), admission ward, initial antimicrobial treatment and clinical diagnosis with specific clinical signs and symptoms recorded at an advanced level.

At **core** level, clinical and laboratory data should be physically linked through two forms printed on either side of the same piece of paper. When entering these data, double entry is preferable to avoid transcription errors, prior to onward electronic transmission at the end of processing. Unique specimen numbers should be assigned to each sample, as well as a unique alphanumeric identifier for the patient episode.

Laboratories should routinely record and report all investigations carried out, including those that are negative. For surveillance purposes, only the first pathogen isolated per patient, in any three month reporting period, should be reported in AMR surveillance. Systematic reporting of data is important to reduce the bias that arises if resistant organisms are over-reported, or reported only if resistant to certain antibiotics.

Sites operating at extended level will capture laboratory data in an integrated electronic system such as WHONET (Appendix A). Clinical and laboratory data should be linked through the unique specimen identification number. WHONET has been developed to facilitate AMR surveillance reporting, but other systems can be used and data specification for aggregated data upload to the GLASS IT platform are available.

3.8 Use of innovative technologies and mobile communications

In high-income countries, innovative technologies for diagnostics, therapeutics and data management are integrated into most health systems, supported by funding streams for research and executive bodies to evaluate and approve new technologies. In LMICs, WHO and other bodies provide support for the implementation of new technologies, and these should be considered by countries developing AMR surveillance.[15] Examples of innovative technologies relevant to AMR surveillance include:[16]

- mobile phone systems for sending microscope images – this could be extended to use of smartphones to share or assess images of disc diffusion assays to confirm zone size
- use of electronic health records (see case study in Bangladesh)
- nucleic acid amplification for TB diagnostics with options for cloud-based reporting
- solar-powered autoclaves
- freeze-drying bacterial isolates for storage (vs freezing at -80°C)

Case study - Bangladesh

Researchers in Bangladesh are using smartphone technology to provide a real-time data capture system for rapid integration of clinical and laboratory data. Clinical workers record data using a smartphone app, which can then be shared with a desktop application used by laboratory workers.

The sample request can be generated remotely and the sample labelled with a unique identifier provided by the system which is instantly associated with the patient and laboratory record.

Use of the smartphone app provides real-time error checking and ensures that all data fields are completed correctly. It also reduces transcription errors, and the need for time-consuming duplicate data entry.

Anonymised data can be processed, stored and shared using cloud storage or FTP servers, reducing the need for on-site server capacity. The system can also allow for automatic upload of data for AMR surveillance analysis.

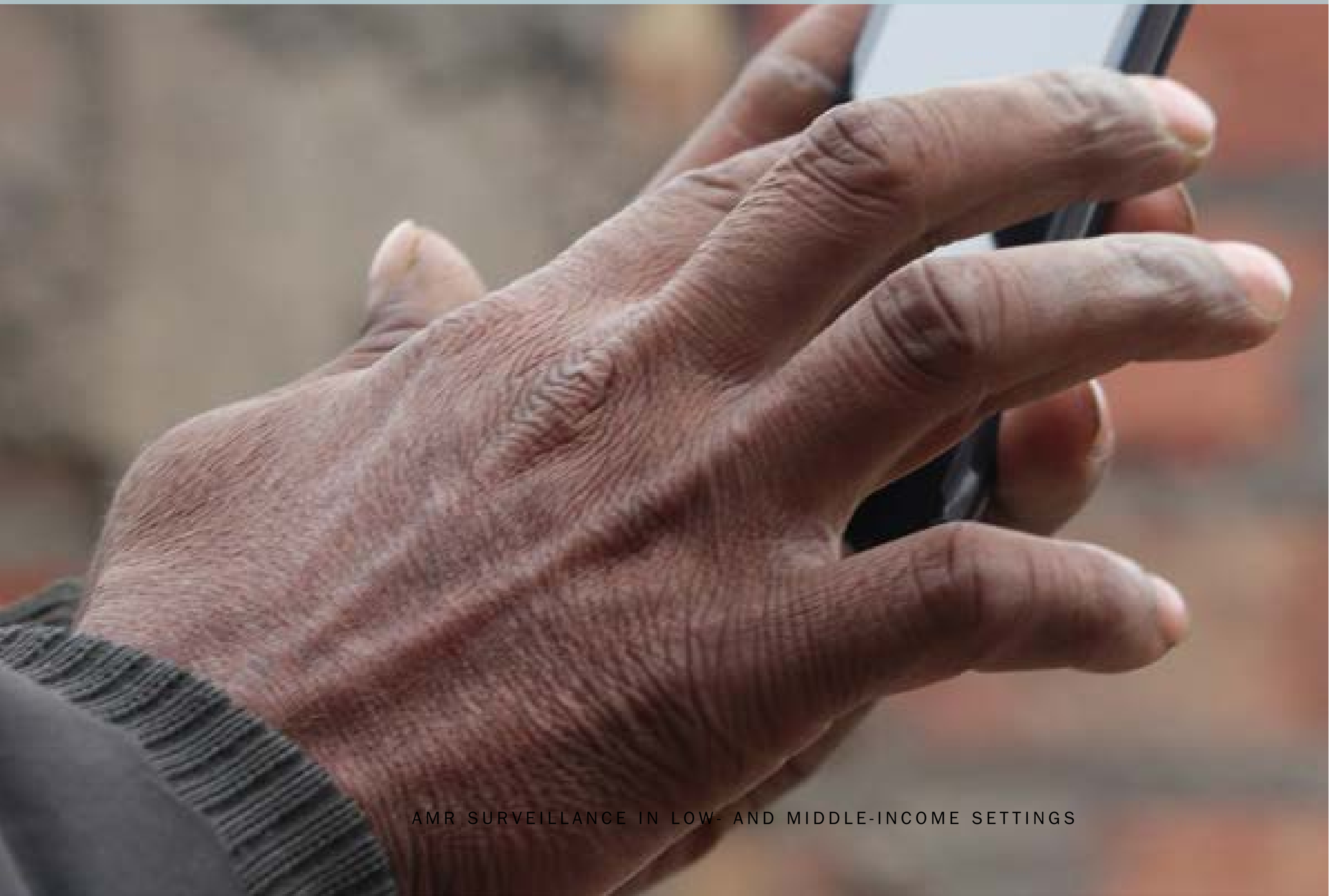

Monitoring, evaluation and development

4

4.1 Quality assurance

Quality assurance (QA) should be led by the national coordinator and technical team in country, in conjunction with external organizations as appropriate. At a **core** level, all procedures should be undertaken according to site-specific SOPs, adapted from national SOPs, and based on these guidelines. Alongside these, quality control (QC) and quality assurance (QA) procedures should be established to ensure that the data produced are accurate and reliable.

4.2 Clinical QA

In a clinical setting, standardisation and investigation should be maintained through quality control procedures, and ensuring completeness of the data and investigations requested through assessment and feedback. To do this, hospital level data on all admission are required to assess, for example, the diagnosis of all patients and whether those with an infectious syndrome were appropriately investigated.

At a **core** level, the quality of clinical sampling and the data acquired should be subject to internal quality assurance assessment through the national coordinator and technical team. At the **extended** level, external assessment would be expected through an independent monitor.

4.3 Laboratory QA

Laboratory QA involves in-house quality control procedures, and internal quality assurance (IQA) and external quality assurance (EQA) assessment.

QA measures include

- the conduct of specimen collection and transportation (e.g. transport times, specimen quality)
- the performance of test procedures, reagents, disks used, media, instruments, and personnel
- test results and documentation

External quality assurance (EQA) is a system for validating laboratory performance using an external, objective agency. EQA is essential for accredited laboratories and, where possible, all laboratories should participate in a formal EQA scheme for all tests performed.

Traditional proficiency testing is considered to be the most cost-effective and useful EQA method. This involves regular (at least annual) dispatch of test isolates to laboratories, to be processed using the normal testing methods by staff who routinely handle such samples. Results are submitted to a central agency, which provides feedback and allows comparison with results from other laboratories (schemes listed in appendix A).

If participation in formal proficiency testing is not possible, adequate EQA may be achieved through a combination of within country retesting/rechecking and internal quality assurance

and control procedures, with periodic external observation of practices and procedures by qualified personnel. This function could be undertaken by the coordinating AMR laboratory.

All laboratories should be engaged in quality improvement (e.g. using the WHO Laboratory Assessment Tool, Appendix A), and should be encouraged to work towards full accreditation (see WHO Stepwise Laboratory Improvement Process Towards Accreditation in the African Region (SLIPTA), Appendix A).

4.4 Data systems QA

Data systems and data management processes should include standard QC measures as described (section 3.7). They should also be subject to quality assessment by the internal National Coordinator and Technical Team and by an external monitor. Evaluation should compare the data system description, the data dictionary and the data report from each site with those from other sentinel sites and other country systems.

4.5 Key Performance Indicators

Key Performance Indicators (KPIs) are used to monitor progress and identify significant problems at sentinel sites where more detailed investigations are needed to understand why the indicators are not being met. The purpose of this investigation is to support sentinel sites to achieve the KPIs. GLASS is developing a monitoring framework for AMR surveillance and provides a sample framework for national KPIs (Appendix A). In-country indicators should be agreed at the inception of AMR surveillance and reviewed annually by the NCC.

Box 2: Summary indicators for a well-functioning sentinel surveillance site

- >80% of all patients admitted with an infectious syndrome are correctly sampled
- >95% of all samples sent for investigation include the physician's clinical diagnosis
- >80% of all blood cultures are of adequate volume (+/- 20% of manufacturer's guideline)
- <10% of samples culture an organisms which is not clinically significant (a contaminant)
- >95% of priority pathogens are correctly identified by the sentinel site laboratory (tested against the gold-standard of the coordinating AMR laboratory or by EQA assessment)
- >95% of the resistance profiles are correctly identified by the sentinel site laboratory (tested against the gold-standard of the coordinating AMR laboratory or by EQA assessment)
- <3 month lag time for reporting all AMR data to the NCC.

Sites will vary in terms of population, geography, and health care facility. However, the criteria given in Box 2 illustrate examples of the criteria which a well-functioning AMR surveillance site would be expected to meet.

4.6 Development of AMR surveillance platforms

4.6.1 Assessing antimicrobial usage

Microbiological data should ideally be interpreted in the context of antibiotic consumption data. This could be done with aggregate data from national wholesale data, or using point prevalence surveys of antimicrobial prescriptions by indication (clinical syndromes), at repeated intervals, for example six-monthly.

4.6.2 Research

The outputs of AMR surveillance should be used to underpin public health policy and, where possible, to answer research questions which will inform our understanding of the emergence and evolution of AMR and help in the development of novel intervention strategies. Research activities, including collaborative scientific work involving surveillance systems in other countries, should be encouraged alongside AMR surveillance.

5 References

1. O'Neill J. Antimicrobial Resistance: Tackling a crisis for the health and wealth of nations 2014. https://amr-review.org/ (accessed 6.6.16).
2. O'Neill J. Tackling Drug-resistant Infections Globally: Final Report and Recommendations 2016. http://amr-review.org/sites/default/files/160518_Final%20paper_with%20cover.pdf (accessed 6.6.16).
3. World Health Organization. Global Action Plan on Antimicrobial Resistance 2015. http://apps.who.int/iris/bitstream/10665/193736/1/9789241509763_eng.pdf?ua=1 (accessed 6.6.16).
4. Horton R. Stumbling around in the dark. *Lancet* 2005; **365**(9476): 1983.
5. World Health Organization. Global Antimicrobial Resistance Surveillance System: manual for early implementation 2015. apps.who.int/iris/bitstream/10665/188783/1/9789241549400_eng.pdf (accessed 6.6.16).
6. Grundmann H, Klugman KP, Walsh T, et al. A framework for global surveillance of antibiotic resistance. *Drug resistance updates : reviews and commentaries in antimicrobial and anticancer chemotherapy* 2011; **14**(2): 79-87.
7. Nsubuga P, White ME, Thacker SB, et al. Public Health Surveillance: A Tool for Targeting and Monitoring Interventions. In: Jamison DT, Breman JG, Measham AR, et al., eds. Disease Control Priorities in Developing Countries. 2nd ed. Washington (DC); 2006.
8. Lee LM, Heilig CM, White A. Ethical justification for conducting public health surveillance without patient consent. *American journal of public health* 2012; **102**(1): 38-44.
9. World Health Organization. Antimicrobial resistance: global report on surveillance 2014. www.who.int/drugresistance/documents/surveillancereport/en/ (accessed 05.05.16).
10. Sane J, Edelstein M. Overcoming Barriers to Data Sharing in Public Health: A Global Perspective. 2015. https://www.chathamhouse.org/sites/files/chathamhouse/field/field document/20150417OvercomingBarriersDataSharingPublicHealthSaneEdelstein.pdf (accessed 27.06.2016).
11. World Health Organization. WHO Guide for the Stepwise Laboratory Improvement Process Towards Accreditation in the African Region (SLIPTA)2015. http://www.afro.who.int/en/clusters-a-programmes/hss/blood-safety-laboratories-a-health-technology/blt-highlights/3859-who-guide-for-the-stepwise-laboratory-improvement-process-towards-accreditation-in-the-african-region-with-checklist.html (accessed 6.6.16).
12. World Health Organisation. Pocket Book of Hospital Care for Children. Problems of the Neonate and Young Infant. Second Edition ed; 2013. p. 45-69.
13. Crump JA, Youssef FG, Luby SP, et al. Estimating the incidence of typhoid fever and other febrile illnesses in developing countries. *Emerging infectious diseases* 2003; **9**(5): 539-44.
14. Editorial. Antimicrobial resistance: the Hydra among us. *The Lancet Infectious Diseases* 2015; **15**(11): 1243.
15. Global Health Technologies Coalition. Advancing research and development to address poverty-related and neglected diseases and conditions. 2014. http://www.ghtcoalition.org/pdf/Summary-paper-advancing-research-and-development-to-address-poverty-related-and-neglected-diseases-and-conditions.pdf (accessed 16.8.16).
16. World Health Organization. Innovative Technologies that Address Global Health Concerns: Outcome of the Call Global Initiative on Health Technologies. 2010. http://apps.who.int/medicinedocs/en/d/Js21569en/ (accessed 6.6.16).
17. World Health Organization. IMAI District Clinician Manual: Hospital Care for Adolescents and Adults. Guidelines for the Management of Illnesses with Limited Resources. 2011. www.who.int/hiv/pub/imai/imai2011/en/ (accessed 16.6.16).
18. World Health Organization. Management of Adolescent and Adult Illness. 2005. http://www.who.int/3by5/publications/documents/imai/en/ (accessed 28.06.16).
19. Singer M, Deutschman CS, Seymour CW, et al. The Third International Consensus Definitions for Sepsis and Septic Shock (Sepsis-3). *JAMA* 2016; **315**(8): 801-10.
20. Lim WS, van der Eerden MM, Laing R, et al. Defining community acquired pneumonia severity on presentation to hospital: an international derivation and validation study. *Thorax* 2003; **58**(5): 377-82.
21. World Health Organization. WHO recommended standards for surveillance of selected vaccine-preventable diseases. *Vaccines and Biologicals* 2003. http://apps.who.int/iris/bitstream/10665/68334/1/WHO_V-B_03.01_eng.pdf?ua=1 (accessed 28.06.2016).
22. Young Infants Clinical Signs Study Group. Clinical signs that predict severe illness in children under age 2 months: a multicentre study. *Lancet* 2008; **371**(9607): 135-42.

6 Acknowledgements

This work was funded by The Fleming Fund through the Department of Health, United Kingdom. It was commissioned from the London School of Hygiene and Tropical Medicine. The first draft was written by Anna C Seale, Nicola C Gordon, Jasmin Islam and Anthony Scott, and re-written with feedback and contributions from an expert working group which met on 6th/7th June 2016 in London, UK.

We would like to thank and acknowledge Charles Penn and Sophie Taysom from the Fleming Fund team and those who attended the expert meeting: Sharon Peacock, Samir Saha, Paul Turner, Rogier Van Doorn, Sam Kariuki, Nick Feasey, Ashika Singh-Moodley, Didem Torumkuney, Tim Peto, Bich-Tram Huynh, Kendall Krause, Li Yang Hsu, Sergey Eremin, Neil Woodford, Nan Shetty, Nienke van de Sande-Bruinsma and Malin Grape. We would also like to thank the following for their written input to the draft guideline: Mark Woolhouse, Anne von Gottberg, John Crump, Chris Parry, Mike Sharland, Nicola Watt, Anders Tegnell, Keith Klugman and Scott Dowell.

7

Appendices

Appendix A: Key documents

Document / procedure	Suggested source	Reference or E-Link
**National Action Plan**	WHO National action plan manual	http://www.who.int/drugresistance/action-plans/manual/en/
**Laboratory Assessment Tool**	WHO Laboratory Assessment Tool	http://www.who.int/ihr/publications/laboratory_tool/en/
**Laboratory accreditation**	WHO Guide for the Stepwise Laboratory Improvement Process Towards Accreditation in the African Region (SLIPTA)	http://www.who.int/tb/laboratory/afro-slipta-checklist-guidance.pdf
**Laboratory quality implementation**	WHO Laboratory Quality Stepwise Implementation Tool	https://extranet.who.int/lqsi/
**Laboratory External Quality Assurance**	UKNEQAS;<br><br>US Association of Public Health Laboratories;	www.ukneqasmicro.org.uk;<br><br>www.aphl.org;
	Canadian Clinical Microbiology Proficiency Testing<br><br>South African National Health Laboratory Service	http://www.cmpt.ca<br><br>http://www.nhls.ac.za/
**Laboratory safety and waste disposal**	WHO laboratory safety manual	http://www.who.int/csr/resources/publications/biosafety/en/Biosafety7.pdf
**Post exposure prophylaxis guidelines**	WHO guidance	http://www.who.int/hiv/pub/guidelines/PEP/en/
**Clinical sampling**	PHE guidelines	https://www.gov.uk/government/collections/standards-for-microbiology-investigations-smi
	WHO guidance	whqlibdoc.who.int/publications/2003/9241545305.pdf
**Sampling processing and identification**	UK-PHE standards for microbiology investigations	https://www.gov.uk/government/collections/standards-for-microbiology-investigations-smi
**Antimicrobial susceptibility testing combinations**	GLASS manual (WHO)	http://www.who.int/antimicrobial-resistance/publications/surveillance-system-manual/en/
**International standards for antimicrobial susceptibility testing**	European Committee on Antimicrobial Susceptibility Testing (EUCAST)	http://www.eucast.org/
	Clinical and Laboratory Standards Institute (CLSI)	http://em100.edaptivedocs.net/Login.aspx
**Data management and reporting**	WHONET software	http://www.who.int/medicines/areas/rational_use/AMR_WHONET_SOFTWARE/en/
**Data security**	Standards to Facilitate Data Sharing and Use of Surveillance Data for Public Health Action (CDC)	http://www.cdc.gov/nchhstp/programintegration/sc-standards.htm
**Key Performance Indicators**	GLASS Manual appendix (WHO)	http://www.who.int/antimicrobial-resistance/publications/surveillance-system-manual/en/
**Monitoring and evaluation of public health surveillance systems**	CDC Guidelines for Evaluating Public Health Surveillance Systems<br><br>Communicable disease surveillance and response systems: Guide to monitoring and evaluating	http://www.cdc.gov/mmwr/preview/mmwrhtml/rr5013a1.htm<br><br>http://www.who.int/csr/resources/publications/surveillance/WHO_CDS_EPR_LYO_2006_2.pdf

Appendix B: Clinical assessment of adults - syndromic diagnoses

Syndrome	Definition[17,18]	Clinical sample(s)
**Sepsis**[19#]<br><br>**ICD-10**<br><br>**R65.2**	Sepsis-related Organ Failure Assessment (quickSOFA) criteria in any patient with suspected infection<br><br>• Respiratory rate ≥22/min<br>• Altered mentation<br>• Systolic blood pressure ≤100 mm Hg	Blood
**Acute bacterial Meningitis**<br><br>**ICD-10**<br><br>**G00-G09**	Onset within hours to days.<br><br>• Fever (≥38.0°C)<br>• Headache<br>• Neck stiffness<br>• Photophobia<br>• Confusion<br>A non-blanching petechial rash may be present in meningococcal meningitis	Blood<br><br>Cerebrospinal fluid if no contraindication to lumbar puncture
**Severe pneumonia**[20]*<br><br>**ICD-10**<br><br>**J09-J18, J20-22**	Cough or difficulty breathing plus at least one of:<br><br>• Very fast breathing (>30 breaths/min)<br>• Temperature 39°C or above**<br>• Pulse 120 or above<br>• SpO_2< 90% (at sea level)<br>• Lethargy<br>• Severe chest pain<br>• Unable to walk unaided<br>• Uncomfortable lying down	Blood
**Acute Severe diarrhoea**<br><br>**ICD-10**<br><br>**A00-A09**	Diarrhoea defined as ≥3 abnormally loose stools per day and lasting <14 days together with evidence of dehydration defined as at least 2 of:<br><br>• Lethargy or unconscious<br>• Sunken eyes<br>• Not able to drink or drinking poorly<br>• Skin pinch returns slowly	Stool
**Severe Soft Tissue Infection**<br><br>**ICD-10**<br><br>**L00-L08**	Ill-defined diffuse swelling of the skin and subcutaneous tissues with redness, tenderness and warmth plus any one of:<br><br>• Systemically unwell<br>• Temperature ≥38.0°C**<br>• Red streaks or tender nodes<br>• Spread to involve significant body surface area<br>• Increased risk of severity or complications<br>• Immunosuppressed<br>• Involvement of genitals, hands, face<br>• Very young or very old	Blood

Syndrome	Definition[17,18]	Clinical sample(s)
**Sexually transmitted infection (Gonorrhoea)**<br><br>**ICD-10**<br><br>**A54.9**	≥1 of the following suggest possible STI:<br><br>• Purulent/mucopurulent discharge +/- cervical friability<br>• Urethral discharge<br>• Dysuria<br>In women PID should be considered if any of the following are present:<br><br>• Lower abdominal tenderness<br>• Fever<br>• Dyspareunia<br>• Uterine bleeding	Urethral swab (male)<br><br>Cervical or high vaginal swab (female)<br><br>Urine
**Pyelonephritis**<br><br>**ICD-10**<br><br>**N10**	Supra-pubic/renal angle tenderness plus any of:<br><br>• Systemically unwell<br>• Fever (≥38.0°C)<br>• Dysuria<br>• Frequency<br>• Haematuria	Blood<br><br>Urine

*The CURB-65 score (**C**onfusion, **U**rea >7 mmol/litre, **R**espiratory rate > 30 breaths/minute, **B**lood pressure (systolic) <90 mmHg or diastolic <60 mm Hg and age ≥**65** years) can also be used to stratify severity of pneumonia.

**Axillary, tympanic or rectal

Risk stratification could be included (https://www.nice.org.uk/guidance/ng51/chapter/recommendations#/identifying-people-with-suspected-sepsis)

Appendix C: Clinical assessment of children – syndromic diagnoses

Syndrome	Definition	Sample
**Severe diarrhoeal disease**<br><br>**(1-59 months)***<br><br>**ICD-10**<br><br>**A00-A09**	Diarrhoea, defined as ≥3 abnormally loose stools in the previous 24 hours. Including, in addition, one of:<br><br>• Sunken eyes, more than normal<br>• Loss of skin turgor<br>• Intravenous rehydration required<br>• Dysentery (diarrhoea with visible blood in stool)<br>• Hospitalization	Stool
**Severe pneumonia**<br><br>**(1-59 months)**<br><br>**ICD-10**<br><br>**J09-J18, J20-22**	Cough or difficulty breathing plus ≥1 of:<br><br>• Central cyanosis<br>• Oxygen saturation <90% (at sea level)<br>• Severe respiratory distress (grunting, in-drawing)<br>• General danger sign (see febrile illness)	Blood
**Severe febrile illness**<br><br>**(1-59 months)**	History of fever (or tympanic temperature of ≥38.0°C) and any general danger sign:<br><br>• Unable to drink or breastfeed<br>• Vomiting everything<br>• Convulsions<br>• Lethargic or unconscious	Blood
**Meningitis[21]**<br><br>**(1-59 months)**<br><br>**ICD-10 G00-99**	*Suspected:* Any person with sudden onset of fever (tympanic temperature of ≥38.0°C) and one of the following signs:<br><br>• Neck stiffness<br>• Altered consciousness<br>• Other meningeal sign	Blood<br><br>Cerebrospinal fluid if no contra-indication to lumbar puncture
**Neonatal possible serious bacterial infection[22]**<br><br>**ICD-10: Neonatal sepsis P36.**	The presence of any one of:<br><br>• Fast breathing (respiratory rate >60 breaths per minute)<br>• Severe chest in-drawing<br>• Hyperthermia >37·5°C<br>• Hypothermia <35·5°C<br>• No movement or movement only on stimulation<br>• Convulsions<br>• Poor feeding	Blood<br><br>Cerebrospinal fluid if no contra-indication to lumbar puncture

Appendix D: National functions for AMR surveillance (core, extended, advanced)

AMR surveillance component		Requirements and standards for core level	Extended level activities*	Advanced level activities**
**Overall aim**		Surveillance data drive national policy and international policy		
**Leadership**	***Data analysis***	National coordinating centre reviews aggregated data with annual report	National coordinating centre reviews aggregated data quarterly with annual report	Real time data presentation (dashboards)<br>Surveillance data are compared to modelled estimates to assess the emergence of resistance and provide early warning for public health action
	Data governance	National standards for data governance and data sharing agreements		
	Assessment of evidence	National coordinating centre reviews aggregated data with expert advice where needed	National coordinating centre liaises with regional network	
	Intervention	Surveillance data drive national policy and international policy		
**Training**	***Clinical***	Training programmes for key staff in core clinical surveillance procedures	Established national training programmes using diverse platforms (e.g. electronic media).<br>Integration of AMR into relevant (undergraduate and postgraduate) programmes.	Functions as a regional centre for international training programmes. Adapts training materials for international use (translation, electronic training packages in different languages)
	Laboratory	Training programmes for key staff in core laboratory surveillance procedures	Established national training programmes using diverse platforms (e.g. electronic media)<br>Integration of AMR into relevant (undergraduate and postgraduate) programmes.	Functions as a regional centre for international training programmes. Adapts training materials for international use (translation, electronic training packages in different languages)
	Data	Training programmes for key staff working in surveillance sites in core data surveillance procedures	Established national training programmes using diverse platforms (e.g. electronic media)	Functions as a centre for international training programmes. Adapts training materials for international use (translation, electronic training packages in different languages)
**Quality Assurance**	***Clinical***	Annual site visit and audit of clinical surveillance at sentinel sites by technical team and national coordinator	Quarterly external audit of clinical data submitted through automated systems and comparison with other sites	
	Sentinel site laboratory	Annual site visit and audit of laboratory standards by technical team and national coordinator	QA assessment of laboratory site to international standards with external accreditation	
	AMR laboratory	Coordinating AMR laboratory participating in EQA and providing internal QA to site laboratories	Coordinating AMR laboratory performs extended testing (e.g. MICs) on a subset of isolates. Collaborates with external partners to investigate exceptional resistance patterns (including WGS).	Provision of whole genome sequencing (WGS) for isolates of interest
	Data	Annual site visit and audit of data systems by technical team and national coordinator	Support for automated sharing of site data for national aggregation	
**Coordinating AMR laboratory**	***Storage of isolates***	Freezer storage (-20°C) of resistant isolates with linkage to paper or electronic database	Reliable freezer storage (-80°C) of resistant isolates with linkage to electronic database#	
	Transport to AMR laboratory	Invasive isolates are transferred to AMR laboratory annually according to local SOPs at acceptable biosafety standards	Invasive isolates are transferred to AMR laboratory quarterly and according to acceptable biosafety standards	

*All core process are assumed in the advanced and extended levels
** All core and extended processes are assumed in the advanced level
# Or other innovative method such as freeze-drying (see section 3.8)

Appendix E: Sentinel site functions for AMR surveillance (core, extended, advanced)

AMR surveillance component		Requirements and standards for core level	Extended level activities	Advanced level activities
**Overall aim**		Surveillance data inform individual care	Surveillance data drive local and national policy (e.g. empiric treatment guidelines, drug procurement) and public health activities	
**Clinical admission assessment and investigation**	***Clinical admission assessment***	Clinical history and examination and investigation based on physician (syndromic) diagnosis.	Systematic clinical history and examination according to clinical algorithms in all patients presenting with suspected infection.	Standardised admission proforma documenting clinical signs and symptoms used to guide diagnosis.
	Clinical data	Clinical data included in (paper) request for laboratory investigation, with unique alphanumeric identifier	Clinical data included in (electronic) request for laboratory investigation, with unique alphanumeric identifier	Linkage of extended clinical data (e.g. vital signs, blood results, outcomes) with laboratory data
	Clinical investigation	Systematic investigation based on physician syndromic diagnosis	Systematic investigation based on clinical findings.	
	Training and quality assurance	Routine training for surveillance SOPs, quality control and Internal Quality Assessment.	External Quality Assessment	Functions as a regional training centre
**Isolate identification and susceptibility testing**	***Sample transport***	Samples transported according to local SOPs	Samples transported according to international biosafety standards	
	Sample registration	Local laboratory paper based data system	Electronic laboratory data system	
	Culture and identification	Automated blood culture system and capacity to identify the relevant priority pathogens according to SOPs	Automated blood culture; CSF, urine, stool and swab culture, identifying all isolates according to SOPs for all priority pathogens.	Automated identification (e.g. MALDI-TOF)
	Susceptibility testing	Use of disc diffusion for blood culture priority pathogens according to SOPs	Use of disc diffusion methods according to SOPs for all species; may include e-tests or broth dilution methods.	Automated identification (e.g. VITEK)
	Training and QA	Routine training for SOPs, quality control and internal QA	External quality assessment	Functions as a regional training centre
**Isolate storage (local) and referral to AMR laboratory**	***Storage of isolates***	Freezer storage (-20°C) of resistant isolates with linkage to paper or electronic database	Reliable (generator back-up) freezer storage (-80°C) of resistant isolates with linkage to electronic database	
	Transport to AMR laboratory	Invasive isolates are transferred to AMR laboratory annually according to SOPs	Invasive isolates are transferred to AMR laboratory quarterly according to international standards for biosafety	
	Training and QA	Routine training for isolate storage, SOPs, quality control and internal quality assessment	External quality assessment	
**Data review**	***Data use***	Anonymised individual data submitted to national coordinating centre and shared regionally and internationally		Automated real time submission of data to national network
	Data linkage	Clinical and laboratory data linked by recording them on the same lab request form	Automated linkage between clinical request data and laboratory data	Automated linkage between clinical and laboratory databases
	Data governance	Data sharing policy and agreements in place in collaboration with the Ministry of Health and/or national public health institute		

AMR SURVEILLANCE IN LOW- AND MIDDLE-INCOME SETTINGS

Appendix F: Minimum level of identification required for the eight priority pathogens

Pathogen	Sample	Identification tests
Escherichia coli	Blood, urine	Gram stain<br>Growth on primary isolation media/selective media<br>Biochemical identification (oxidase, indole, urease and carbohydrate fermentation tests)<br>Serological typing (agglutination testing for *Salmonella/ Shigella* spp.)
Klebsiella pneumoniae	Blood, urine	
***Salmonella* spp.**	Blood, stool	
***Shigella* spp.**	Stool	
Neisseria gonorrhoeae	Urethral swab, cervical swab,	Gram stain<br>Growth on selective media<br>Biochemical identification (oxidase, carbohydrate utilisation tests)<br>Detection of pre-formed enzymes<br>Immunological reactivity with gonococcal specific antibodies
Acinetobacter baumannii*	Blood	Gram stain<br>Biochemical identification (oxidase, catalase tests)<br>Non-haemolysis<br>Acidification of glucose
Staphylococcus aureus	Blood,	Gram stain<br>Biochemical identification (catalase test, coagulase test, growth on selective indicator media e.g. Chromagar)
Streptococcus pneumoniae	Blood, CSF	Gram stain<br>Colonial appearance (draughtsman colonies)<br>Biochemical identification (catalase, bile solubility test)<br>Optochin sensitivity

*Accurate speciation of *A. baumannii* is rarely possible without the use of genetic methods. Isolates may be identified to genera level and reported as *A. baumannii-calcoaceticus* complex, with speciation performed if required by the AMR laboratory

Appendix G: Pathogen-antimicrobial combinations (table from GLASS manual)

Pathogen	Antibacterial class	Antibacterial agents that may be used for AST [a,b]
Escherichia coli	sulfonamides and trimethoprim	co-trimoxazole
	fluoroquinolones	ciprofloxacin or levofloxacin
	third-generation cephalosporins	ceftriaxone or cefotaxime and ceftazidime
	fourth-generation cephalosporins	cefepime
	carbapenems[c]	imipenem, meropenem, ertapenem or doripenem
	polymyxins	colistin[f]
	penicillins	ampicillin

Pathogen	Antibacterial class	Antibacterial agents that may be used for AST [a,b]
Klebsiella pneumoniae	sulfonamides and trimethoprim	co-trimoxazole
	fluoroquinolones	ciprofloxacin or levofloxacin
	third-generation cephalosporins	ceftriaxone or cefotaxime and ceftazidime
	fourth-generation cephalosporins	cefepime
	carbapenems[c]	imipenem, meropenem, ertapenem or doripenem
	polymyxins	colistin[f]
Acinetobacter baumaannii	tetracyclines/glycylcycline	minocycline/tigecycline
	aminoglycosides	gentamicin and amikacin
	carbapenems[c]	imipenem, meropenem, ertapenem or doripenem
	polymyxins	colistin[f]
Staphylococcus aureus	penicillinase-stable beta-lactams	oxacillin[e] or cefoxitin[d]
Streptococcus pneumoniae	penicillins	oxacillin[e]<br>penicillin G
	sulfonamides and trimethoprim	co-trimoxazole
	third-generation cephalosporins	ceftriaxone or cefotaxime
***Salmonella* spp.**	fluoroquinolones<br>third-generation cephalosporins<br>carbapenems[c]	ciprofloxacin or levofloxacin<br>ceftriaxone or cefotaxime and ceftazidime<br>imipenem, meropenem, ertapenem or doripenem
***Shigella* spp.**	fluoroquinolones<br>third-generation cephalosporins<br>macrolides	ciprofloxacin or levofloxacin<br>ceftriaxone or cefotaxime and ceftazidime<br>azithromycin
Neisseria gonorrhoeae	third-generation cephalosporins	cefixime<br>ceftriaxone
	macrolides	azithromycin
	aminocyclitols	spectinomycin
	fluoroquinolones	ciprofloxacin
	aminoglycosides	gentamicin

a The listed substances are priorities for surveillance of resistance in each pathogen, although they may not be first-line options for treatment. One or more of the drugs listed may be tested.

b ≥1 of the drugs listed may be tested in countries. S, I, R and nominator and denominator data for each reported separately.

c Imipenem or meropenem is preferred to represent the group when available.

d Cefoxitin is a surrogate for testing susceptibility to oxacillin (methicillin, nafcillin); the AST report to clinicians should state susceptibility or resistance to oxacillin.

e Oxacillin is a surrogate for testing reduced susceptibility or resistance to penicillin; the AST report to clinicians should state reduced susceptibility or resistance to penicillin.

f Microbroth dilution is recommended for colistin susceptibility testing for Gram negatives: this would be an extended level function.

London School of Hygiene & Tropical Medicine
Keppel Street
London WC1E 7HT
United Kingdom

Improving health worldwide

www.lshtm.ac.uk
