# Supplementary material for: AMR Surveillance in low and middle-income settings - A roadmap for participation in the Global Antimicrobial Surveillance System (GLASS)
Source: Wellcome Open Res. 2017 Sep 26;2:92. [Version 1] doi: 10.12688/wellcomeopenres.12527.1 (PMC5645727; doi:10.12688/wellcomeopenres.12527.1)
Supplement: Peer review history

# Peer review: Referee response for version 1

**DOI:** 10.21956/wellcomeopenres.13565.r26408

**Published:** 2017-10-16

**Authors:** Ben Amos, Cyril Buhler

**Affiliations:** 1 Mott MacDonald, London, UK; 2 ORDiagnostics, Paris, France

This article by Anna Seale *et al.* and the accompanying roadmap offers a comprehensive framework to build a national human health AMR surveillance system. It is clearly presented and is a very helpful guide for the many low and middle income countries that are setting up surveillance systems at present. The Fleming Fund, which sponsored this study, is using the roadmap in discussions for support to countries. There is a need for similar help in other areas of the surveillance of antimicrobial resistance in animals and the environment, antimicrobial use, and in linking all these areas together as a one health approach. 

 The roadmap proposes a sentinel site system at three levels core, extended and advanced. The core level is intended to be the minimum acceptable standard and setting this standard is very helpful. The extended and advanced levels give suggestions for how to improve the quality of the surveillance. As many of these suggestions can be implemented individually the authors should consider representing these as a progression in each area rather than as discrete levels. For example surveillance systems may choose to use software such as WHONET for entry and analysis of antibiotic susceptibility data at even core level facilities. 

 The choice of blood culture as the starting point for surveillance is understandable due to the seriousness of sepsis. Blood culture is not however the easiest sample type to begin with from both clinical and laboratory perspectives. It is expensive and technically challenging, the rate of positivity is often low. The authors should consider to recommend that countries also start with one or more other sample type at core level. Both urine and stool are easier and cheaper to deal with and have a limited range of reportable species within GLASS. Surveillance of these would produce useful data much faster than blood culture. 

 The value of linking surveillance with research could be further emphasised. Many countries have proficient research organisations that are eager to be involved and have existing capabilities and equipment (for example next gen sequencers) that can be used to enhance the usefullness of the surveillance data. Some countries will choose to run surveillance at site with existing capacity such as demographic surveillance which are general run by research groups. Linkage with academic institutions will also be useful in encouraging full exploration and use of the data that comes out of the surveillance, an area in which there is little guidance.

We have read this submission. We believe that we have an appropriate level of expertise to confirm that it is of an acceptable scientific standard.

# Peer review: Referee response for version 1

**DOI:** 10.21956/wellcomeopenres.13565.r26410

**Published:** 2017-10-16

**Authors:** Fatima Serhan

**Affiliations:** 1 Expanded Program on Immunization, World Health Organization (WHO), Geneva, Switzerland

Thanks for providing me opportunity to review the paper on roadmap for low and MIC to participate in AMR surveillance system.

 The publication is of excellent value to guide countries on implementation steps for AMR surveillance and gives a good summary of the necessary steps and bodies needed for successful implementation. Based on my experience in coordinating WHO surveillance systems for bacterial pathogens, bacterial capacities in many developing countries are limited and often antibiotic use prohibits bacterial culture and interferes with the right clinical treatment. I encourage the authors to highlight importance that MOH and surveillance coordinators for AMR to align efforts with existing long standing surveillance systems and reinforce collaboration with the sentinel sites surveillance and reference laboratories for other bacterial networks at both country and regional levels in a an integrated rather than vertical approaches to strengthen surveillance systems.

 I would also like to draw the attention of the authors to the facts that the no timelines for implementation of action plan at country level are proposed, and encourage them to add something on that matter.

 Estimation of costing of surveillance activities described at country level would be an asset to help countries while developing of national action plan. Reference to donors who will be willing to support the work especially in low income countries would also help.

I have read this submission. I believe that I have an appropriate level of expertise to confirm that it is of an acceptable scientific standard.

# Peer review: Referee response for version 1

**DOI:** 10.21956/wellcomeopenres.13565.r26409

**Published:** 2017-10-11

**Authors:** Richard A. Adegbola, Stephen K. Obaro

**Affiliations:** 1 GlaxoSmithKline (GSK), Wavre, Belgium; 2 University of Nebraska Medical Centre, Omaha, NE, USA

This is a well written article. The proposed conceptual frame work for monitoring AMR in Low and Middle-Income Countries (LMIC) is comprehensive and detailed. There are however, a few fundamental issues that should be considered to improve the quality of data, host country compliance and benefit, and sustainability of the programme. 

1. In most LMIC settings, there is unrestricted use and access to a broad range of antibiotics. The unrestricted use is largely driven by the absence of microbiology diagnostic laboratories both at Government and public health facilities. At these locations access cannot be ethically restricted if health care facilities are not equipped with microbiology diagnostic laboratories with trained personnel to provide quality diagnostic services at affordable cost to the general population. Provision of such facilities will effectively guide and restrict antibiotic use and enhance the quality of AMR surveillance data. Thus, the approach to implementing effective surveillance for AMR in LMIC settings need to be 3-pronged:
  a) Strengthening microbiology diagnostic services at all tiers of both private and Government run health care services
  b) Use of laboratory data to guide and restrict antibiotic use
  c)Creating effective and sustainable AMR surveillance.
2. Since ultimately the goal of setting up AMR surveillance includes monitoring the economic impact, it is important to actively incorporate plans for monitoring clinical outcomes in any framework for LMIC settings. At most locations, it is not customary to return to a health care facility after an acute illness, and record keeping is often less than optimal, thus acquisition of clinical outcome data may warrant a proactive plan with home visits or an outreach team that is based in the community for early, intermediate or late outcomes. 
3. A key component of AMR surveillance is timely dissemination of surveillance data to relevant stakeholders. The programme should include plans to extend timely dissemination of surveillance data beyond scientific and medical community to include the general public, policy-makers and governments. This will be crucial to raising the levels of awareness of AMR and local ownership in order to ensure sustainability of the programme in LMICs.

We have read this submission. We believe that we have an appropriate level of expertise to confirm that it is of an acceptable scientific standard.
